# Making sense of DialysisConnect: a qualitative analysis of stakeholder viewpoints on a web-based information exchange platform to improve care transitions between dialysis clinics and hospitals

**DOI:** 10.1186/s12911-021-01415-y

**Published:** 2021-02-09

**Authors:** Ann E. Vandenberg, Bernard G. Jaar, Kyle P. James, Janice Lea, Christopher O’Donnell, Tahsin Masud, Rich Mutell, Laura C. Plantinga

**Affiliations:** 1 Emory University School of Medicine, Atlanta, GA USA; 2 Johns Hopkins University, Baltimore, MD USA; 3 Apex Health Innovations, Williamsburg, VA USA

## Abstract

**Background:**

U.S. hospitals and dialysis centers are penalized for 30-day hospital readmissions of dialysis patients, despite little infrastructure to facilitate care transitions between these settings. We are developing a third-party web-based information exchange platform, DialysisConnect, to enable clinicians to view and exchange information about dialysis patients during admission, hospitalization, and discharge. This health information technology solution could serve as a flexible and relatively affordable solution for dialysis facilities and hospitals across the nation who are seeking to serve as true partners in the improved care of dialysis patients. The purpose of this study was to evaluate the perceived coherence of DialysisConnect to key clinical stakeholders, to prepare messaging for implementation.

**Methods:**

As part of a hybrid effectiveness-implementation study guided by Normalization Process Theory, we collected data on stakeholder perceptions of continuity of care for patients receiving maintenance dialysis and a DialysisConnect prototype before completing development and piloting the system. We conducted four focus groups with stakeholders from one academic hospital and associated dialysis centers [hospitalists (n = 5), hospital staff (social workers, nurses, pharmacists; n = 9), nephrologists (n = 7), and dialysis clinic staff (social workers, nurses; n = 10)]. Transcriptions were analyzed thematically within each component of the construct of coherence (differentiation, communal specification, individual specification, and internalization).

**Results:**

Participants differentiated DialysisConnect from usual care variously as an information dashboard, a quick-exchange communication channel, and improved discharge information delivery; some could not differentiate it in terms of workflow. The purpose of DialysisConnect (communal specification) was viewed as fully coherent only for communicating outside of the same healthcare system. Current system workarounds were acknowledged as deterrents for practice change. All groups delegated DialysisConnect tasks (individual specification) to personnel besides themselves. Partial internalization of DialysisConnect was achieved only by dialysis clinic staff, based on experience with similar technology.

**Conclusions:**

Implementing DialysisConnect for clinical users in both settings will require presenting a composite picture of current communication processes from all stakeholder groups to correct single-group misunderstandings, as well as providing data about care transitions communication beyond the local context to ease resistance to practice change.

**Supplementary information:**

The online version contains supplementary material available at 10.1186/s12911-021-01415-y.

## Background

U.S. end-stage renal disease (ESRD) patients receiving maintenance dialysis are hospitalized frequently (1.8 admissions annually), and more than 35% of these hospitalizations are followed by costly 30-day readmissions [1, 2]. In response, the Centers for Medicare & Medicaid Services (CMS), which covers most U.S. dialysis treatment, penalizes both hospitals and dialysis clinics for readmissions among these patients [3–5]. Most dialysis clinics and hospitals do not belong to the same healthcare system or share the same electronic health record (EHR) [6]. Without interoperability capabilities across EHRs, these settings cannot seamlessly exchange information to facilitate care transitions for hospitalized dialysis patients [6–8]. As a working solution, we are developing a web-based information exchange platform, DialysisConnect, which will allow clinicians in both settings to exchange information about dialysis patients during hospitalization. This health information technology solution, which circumvents the issues of direct communication within our multi-payer, multi-EHR system, could serve as a flexible (i.e., EHR-agnostic) and relatively affordable solution for dialysis facilities and hospitals across the nation who are seeking to serve as true partners in the improved care of dialysis patients.

Using a modified Agile Development process, our development team is designing a web-based secure HIPAA platform based on the Cloud infrastructure from U.S. Oracle Corporation (Redwood City, California). The Oracle Application Express development environment can accommodate iterative modifications from pre-implementation discussions with potential stakeholders, during user testing, and during piloting in real time clinical care. The four participating dialysis clinics have a secure application programming interface (API) to upload their patient census on a scheduled basis into the DialysisConnect platform. The purpose of the auto-load API is to provide an initial data load to avoid double-data entry and to provide commonly requested data such as patient demographic and medical information. Information additional to the auto-load will need to be requested by system users and sent by the other setting. The DialysisConnect application will not interface to local EHR systems for this study, although this capability could be built into later versions.

We recognize that regional healthcare information exchanges (HIEs) were created to address some of the gaps we plan to bridge with DialysisConnect. In our region, the Georgia Health Information Network is a public–private collaborative that works to close the patient information gap across healthcare settings [9]. However, HIEs generally do not include dialysis facilities. Furthermore, while EHR information can be shared and e-mails sent, communication may not be timely, there may be no context for the information shared, and the information may not include what the provider is seeking (*e.g*., information that may be in medical notes rather than in the EHR). Evidence regarding better clinical outcomes with HIE use is mixed and based on non-rigorous methods [10]. Finally, different HIEs may have different products, costs, and requirements, which make HIEs infeasible as a solution across regions.

DialysisConnect is based on the same platform of a similar application called the Transplant Referral Exchange (T-REX) that is being used across all nine transplant centers in three southeastern U.S. states (North Carolina, South Carolina, and Georgia) and by clinics in the two largest national dialysis organization to facilitate kidney transplant referral. T-REX has been accepted by dialysis and transplant providers. Thus, we expected that dialysis providers would be willing to test the technology in a similar format. Furthermore, DialysisConnect in its proposed form will be flexible, efficient, and responsive to the needs of the users/providers, thus allowing us first to determine the general effectiveness and sustainability of the communication process itself, before building a more expensive EHR-interfacing system.

As part of this larger study, we are piloting DialysisConnect at one urban academic hospital and four associated dialysis centers, which have separate EHRs. The hospital’s providers will have access to automatically uploaded basic demographic and clinical information about their dialysis patients and have clinic and nephrologist contact information. The system allows hospitalists to request additional medical information from dialysis clinics to improve hospital care. It allows them to submit discharge information ahead of the hospital discharge summary so that dialysis centers will be informed of changes to a dialysis patient’s care ahead of their next dialysis sessions. Dialysis providers can also request further information from hospitals. Clinicians in both settings can exchange additional information through the system’s communication channel.

Measuring the success of DialysisConnect is predicated on its successful implementation. As theorized by the Normalization Process Theory (NPT) [11], complex technological interventions will only be effective if they are normalized into the workflow of the user, and for this to happen they must be coherent to users. Prior to the ongoing pilot, we held focus groups with potential user groups both to solicit feedback and revisions to the system and also to evaluate the perceived coherence of DialysisConnect to shape implementation messaging. Here, we report on the latter aim.

## Methods

As part of a hybrid effectiveness-implementation study [12] guided by NPT, we collected data on stakeholder perceptions of continuity of care for ESRD patients receiving maintenance dialysis and a DialysisConnect prototype before completing development and piloting the system. We purposively recruited participants from four groups of potential users: hospitalists, hospital staff, dialysis clinic nephrologists, and dialysis clinic staff. Focus groups were 90-min and guided by the same interview guide (Table 1). Following a preliminary discussion, a 13-min video simulation of DialysisConnect was shown to the participants (see Additional File 1 for still images from this video). The interview was divided into three phases: Phase 1, an open discussion of communication between dialysis clinics and hospitals; Phase 2, a discussion of the prototype response discussion; and Phase 3, a summary discussion. All focus groups were held in March 2019. Each focus group was digitally recorded, transcribed, and coded using grounded theory methods (open coding and focused coding) [13, 14].

**Table 1:** DialysisConnect focus group guide

Initial open ended discussion
What comes to mind when you think about communicating with the dialysis clinic about a dialysis patient?
What communication about the patient do you typically have with the [dialysis clinics/hospital] upon hospital admission? Probe: Information needed and received Personnel who exchange information Documentation Time involved
What communication about the patient do you typically have with the [dialysis clinics/hospital] upon hospital discharge? Probe: Information needed and received Personnel who exchange information Format(s) of communication Time involved and timeliness
What is your opinion about the current communication process between hospitals and dialysis clinics? Probe: What is working well that should not be changed? What is problematic about the process? How well does the current process serve patients? How could the process be improved?
Prototype response discussion
What are your initial reactions to the video you saw?
Now I’d like to get your thoughts about each part of the flow process in the prototype Logging in Inputs to find dialysis patient Information in patient profile Initial hospital reason Request to dialysis clinic to upload needed information and documents Quick visual cue panel Process for reminding dialysis clinic if requested information or documents is not received Discharge information for hospital side to upload Personnel and roles
Summary discussion
What would the addition of DialysisConnect mean for hospital providers? How would it be different from current practice?
[Summary of discussion.] Is this an adequate summary of what we discussed? Is there anything else you would like to add?

NPT was chosen as a framework for the study in order to assess the normalization potential of DialysisConnect. NPT is ideal for pragmatic research, which seeks to identify sustainable, practical solutions to problems in healthcare delivery, and is particularly suited to the proposed technology-based intervention. NPT has been used to qualitatively evaluate a diverse set of complex interventions within healthcare, for example telehealth in respiratory medicine and a new discharge planning process [15, 16]. The emphasis of NPT is on the mental and physical actions of the users of a new technology or intervention that result in successful implementation through behavior change; the four core constructs are coherence (making sense of the problem), cognitive participation (building a new community of practice), collective action (operationalization of practice), and reflexive monitoring (appraisal of practices) [11]. The work of implementation begins with coherence, or how people invest the new technology with meaning; studies have demonstrated that perceived coherence is important for staff buy-in and participation [6, 17–20]. The construct of coherence itself has four components: *differentiation*, participants’ understanding of how the technology is distinct from the current approach; *communal specification*, participants’ assessment of the value of the technology; *individual specification*, individual team members’ understanding of specific tasks and responsibilities within their role in relation to the technology; and *internalization*, participants’ belief in how the technology will help them or their patients in their work. Following open coding [13], transcripts were grouped under NPT coherence components and then examined thematically within and across stakeholder groups [14]. Additional File 2 details our methods according to the Consolidated Criteria for Reporting Qualitative Research [21].

## Results

The characteristics of our focus groups are described in Table 2.

**Table 2:** Focus group participant characteristics

Characteristics	Hospitalists (n = 5)	Hospital staff (n = 9)	Nephrologists (n = 7)	Dialysis clinic staff (n = 10)
Age: mean (range)	41 (31–58)	49 (27–65)	49 (39–63)	50.8 (41–60)
Sex (F): count (%)	5 (100%)	9 (100%)	2 (28.5%)	10 (100%)
Ethnicity: count (% not Hispanic)	5 (100%)	9 (100%)	7 (100%)	10 (100%)
Race: count (%)				
White	2 (40%)	2 (22%)	2 (28.5%)	2 (20%)
Black	1 (20%)	7 (78%)	2 (28.5%)	8 (80%)
Other	2 (40%)	–	3 (42.9%)	–
Clinical role: count (%)				
Attending	5 (100%)	–	4 (57.1%)	–
Fellow	–	–	3 (42.9%)	–
Pharmacist	–	1 (11%)	–	–
Nurse practitioner	–	3 (33%)	–	1 (10%)
Registered nurse	–	4 (44%)	–	2 (20%)
Social worker	–	1 (11%)	–	6 (60%)
Dietician	–	–	–	1 (10%)
Years of practice: mean (range)	9.2 (5–13)	21 (4–37)	12.4 (1–25)	17.5 (3–31)
Years of treating dialysis patients: mean (range)	10.4 (8–14)	12 (1.5–33)	13.1 (1–30)	8.45 (1.5–18)
Percent of clinical work time: % (range)	74% (50–100%)	87% (40–100%)	81.4% (40–100%)	54.5% (4–100%)

The results of our thematic analysis are presented by coherence construct component below and in Table 3 with illustrative quotes.

**Table 3:** Themes by coherence constructs for DialysisConnect

Coherence construct	Theme	Illustrative quotes
Differentiation: Participants’ understanding of how the technology is distinct from the current approach	Differentiating as a communication channel	“All I really cared about was the fact that you could exchange like ‘Hey, I want a med rec, or I want XYZ.’”—Hospitalist “I, honestly, would probably try to text and say, ‘Hey, can you just tell me what their potassium was yesterday?’ instead of asking ‘Can you upload all their labs?’—Hospitalist nurse practitioner
	Differentiating as an information dashboard	“When I log in, am I gonna see a dashboard like this [DialysisConnect] or do I have to search the patient, then one patient comes up?”—Hospital registered nurse “We sort of get an idea about how many patients or what percent of patients are hospitalized at any given time, which may be pretty high.”—Nephrologist “You get all of your patients’ information all at once. The alerts will tell you these patients are hospitalized, as opposed to looking them up one by one.”—Dialysis clinic social worker
	Differentiating as expedited discharge information delivery	“Having DialysisConnect is better than not having it at all, specifically for communicating discharge.”—Hospitalist “To me this is an extra step to try to complete my discharges, because I [currently] don’t have to do my discharges on the same day”—Hospital nurse practitioner “The discharge summary depends on when the hospitalist is able to complete it…Other information, like the labs, medications, any access-related followup, can be done right away”—Nephrology fellow (working in hospital) “I think it’ll be very helpful, compared to we don’t really have anything right now, if hospitals are able to give us some information.”—Dialysis clinic social worker
	Not differentiating meaningfully	“I totally rely on [name of the healthcare system’s] nephrologists, and we talk with them every single day. But when you are asking us to do extra and extra things, it creates lots of distraction.”—Hospitalist “They [dialysis clinic staff] don’t take the initiative to pick up the phone. Why would we think they’re gonna take the initiative to input things [into DialysisConnect] when we need them?”—Hospital nurse practitioner “It’s another layer of bureaucracy. Because we’re supposed to check in the EMR once a day, and this is another thing to check.”—Nephrologist
Communal specification: Participants’ assessment of the value of the technology	Perceiving too much effort to benefit within local health system	“I can’t imagine somebody would be tasked with having to enter a reason for admission when they don’t really have any specific benefit from that. I think the whole point is to help us to connect. But I think we’re going to contaminate [DialysisConnect] with so much information I think we can find in the chart that I think that it becomes very redundant.”—Hospitalist “It’s too much going on in the day and we’re seeing too many patients. We don’t have time to sit and say, ‘Let me look up this system. Let me go through this system.’ Because we already have our own system that I’m already going back through.”—Hospital nurse practitioner “Right now we just call the dialysis unit, and we get information that way. I don’t have to wait for documents to be scanned and sent to me. That’s more effective than me trying to wait for something.”—Nephrology fellow (working in hospital) “I’ll second that.”—Nephrologist
	Perceiving effort to benefit as worthwhile within broader health system	“I understand it in the big scope of things.”—Hospitalist “There’s great potential outside of [name of the healthcare system]. But within [name of the healthcare system] there may not be.”—Nephrologist “We do have PowerChart, so that is helpful. We’re able to obtain some information, and I guess for the outside hospitals we’re just kind of like, whoa. We don’t have any information.”—Dialysis clinic social worker “We really don’t have any”—Dialysis clinic nurse practitioner
Individual specification: Individual team members’ understanding of specific tasks and responsibilities in relation to the technology	Delegating tasks and responsibilities to others	“The Emergency Room has resources”—Hospitalist “Your best bet would be to have one person whose responsibility it is to make sure that discharge is complete. Cause I feel like if you have too many people touching a dashboard, it’s gonna be off. What I’m concerned about is … if the day is busy, I’m not gonna keep pulling this up. It’s a whole separate system to log into.”—Hospital nurse practitioner “So it wouldn’t be the physician then, right? I mean, if one of my patients gets admitted to the hospital and the person on the hospital side wants information about it, going to me isn’t going to help. So that would have to be directed to one of the staff at the clinic.”—Nephrologist “I say the AAs (dialysis administrative assistants) should be handling these requests”—Dialysis clinic registered nurse, with unanimous agreement from group
	Doubting that others can or will participate	“A lot of this nice uploading and putting comments, you’re being nice, but if you’re really busy, this is not what somebody’s going to do.”—Hospitalist “Who is going to be the gatekeeper and who is going to pay that gatekeeper? That’s gonna be the bottom line. Who’s gonna pay somebody to man a system that isn’t going to benefit the hospital wide?”—Hospital registered nurse “The charge nurse, she’s busy with all sorts of other things. So I think it’s going to be difficult for her to do all of that”—Nephrology fellow “At the dialysis clinic, we can assign someone’s job at doing it and they would have to do it, and that would be the end of it. Ok. So that could be done. I don’t think we can tell hospital personnel what to do in terms of their responsibility there.”—Nephrologist
Internalization: Participants’ belief in how the technology will help them or their patients in their work	Comparing to other technologies, unfavorably or favorably	“It would be great to have a unified system so that we don’t have to create sub systems”—Hospitalist “All the clinics in New Orleans when Katrina hit were under water. But all Davita clinics, they all, every night, everything was pushed up onto the Tacoma server. I sat in Houston and with my login could actually log into the clinic although the clinic was under water because it was going up on the server in Tacoma.”—Hospital nurse practitioner “Can we take staff out and seamless get those last flow sheets, labs and medications, automatically uploaded for these patients?”—Nephrology fellow “They’re already doing it now, recently for the past year”—Nephrologist [misunderstanding] “It is like that with T-REX [transplant referral management technology similar to DialysisConnect]. I think it’s easy”—Dialysis clinic social worker
	Embracing idea for long-term care of dialysis patients	“We need to do this. We need to communicate clearly. No doubt about that…That is why we are here. We want it to be better for our patients and these people are super complicated and very sick.”—Hospitalist “I see this as being positive more in physician-to-physician communication regarding a long-term care of a patient”—Nephrology fellow DialysisConnect would mean “Just giving more continuity of care”—Dialysis clinic social worker

### Differentiation

Participants differentiated DialysisConnect favorably from usual care in various ways. Hospitalists and hospital staff, who acknowledged that they lacked baseline information on admitted dialysis patients, characterized DialysisConnect primarily in terms of its communication channel (the ability to text a contact at the other setting within the system). These groups noted the advantage as having the patient’s dialysis clinic and contact information for the first time in the automated file feed from dialysis clinics. The exchange potential of DialysisConnect stood out as more important than having a complete array of information.

All groups except the hospitalists differentiated DialysisConnect as an information dashboard displaying all dialysis patients admitted to the hospital; individual entries display identifying and clinical information, and the status of information requests and delivery about them. A dialysis clinic social worker saw time efficiencies in this array, noting the potential to generate useful reports on trends in dialysis patient hospitalizations. A nephrologist grappled with the scope of DialysisConnect involvement by estimating that about 10% of dialysis clinic patients (or about 75 patients) could be displayed at any one time. The concept of a dashboard raised questions for some hospital staff about how responsibilities for these patients would be divided, and one wanted an individual dashboard of just her patients.

All groups also differentiated DialysisConnect as expedited discharge information delivery, including DialysisConnect’s potential to deliver some discharge instructions directly from hospitals to dialysis clinics without the problems of faxing or involving an information transfer center. Hospitalists noted the lack of an established channel for sending antibiotic information to dialysis clinics and lack of confirmation on follow through when information was sent. A nephrologist and a hospital registered nurse (RN) saw potential to send labs, medications, and follow-ups to the clinics ahead of patient’s return to dialysis without waiting for a provider to submit a discharge summary. One hospital nurse practitioner (NP) saw this potential negatively, as requiring providers to do more discharge reporting under a stricter timeline.

All groups except the dialysis clinic staff (with experience with a similar system) struggled at times to meaningfully differentiate the DialysisConnect process from current practice. They characterized the status quo as based on unreliabilities (e.g., patients carrying information to the other setting, patient or nephrologist memory, staff responding to requests). However, with DialysisConnect, they identified other potential workflow unreliabilities, including the need for someone to initiate patients into the system, goodwill and mindfulness to participate, and waiting periods between information requests and information uploads and notifications while still needing to provide care. These potential unreliabilities suggested that the perceived advantage of DialysisConnect over the *status quo* was equivocal.

### Communal specification

In assessing the value of DialysisConnect, groups weighed perceived effort needed to use the system against perceived benefits. All but the dialysis clinic staff group anticipated effort of incorporating DialysisConnect into their workflow to outweigh benefit as currently presented. Hospitalists saw purpose in the communication channel but not in requesting information from dialysis clinics and waiting for it to arrive and did not always see the need to expedite discharge information. Some hospital staff were under the impression that discharge summaries were already seamlessly reaching dialysis clinics. A hospitalist could not see a benefit for entering the patient’s hospitalization reason into the system. A nephrology fellow characterized manual uploads as not only labor intensive but also potentially error prone. A nephrologist noted that the sheer numbers of hospitalized dialysis patients evoked “the daily burden of having to do this.” Almost all groups wanted additional automation.

All groups suggested system redesigns for improved workflow efficiency. Hospitalists wanted dialysis clinics to initiate patients into the DialysisConnect system in order to have information waiting for them upon patient admission. This suggestion was based on the impression that dialysis patient admissions came directly from dialysis clinics “95% of the time.” This number contrasted with 10% estimated by a dialysis social worker, but dialysis staff agreed that entering information into the system for hospitals for this subset was prudent. Nephrologists pointed to the required initiation of the system at the hospital setting as a flaw; without this initiation, dialysis clinics would not be able to request discharge information. Dialysis clinic staff requested anticipatory uploading of discharge information at or before discharge.

Current system workarounds, namely nephrologists’ joint privileges at both settings, were acknowledged as the reason further effort would not yield benefit. The effort-value balance changed when considering hospitals and clinics outside of their healthcare system where those workarounds did not exist. Nephrologists repeatedly asked about inclusion of these entities for which contact information and relationships were lacking and delays for receipt of hospital discharge summaries could take weeks. They acknowledged that requesting records from those hospitals was not routine, and staff described a laborious process for requesting these documents, sometimes without response. Groups acknowledged value of the current pilot project as a bridge to wider dissemination outside of the healthcare system.

### Individual specification

From communal specification, it was clear that using the system would require people to upload and download information into the system in a new process. The groups were well aware that this task would fall to specific people. In addition, providers would have to be responsible for being aware of this exchange through alerts. All groups attempted to negotiate the delegation of DialysisConnect tasks to personnel besides themselves. The hospitalists and nephrologists delegated admission tasks to emergency department staff and to the dialysis clinic staff when patients were being sent from the clinics. One hospitalist saw the system first as “nurse driven” and then suggested a secretary as “nurses have a lot of things to do.” While a hospital staff member and RN accepted some of the new tasks as consistent with what they currently do, another RN objected that “more documentation, it can be a deterrent” and others concurred. The staff member then reversed course and questioned the need for a new system at all. While the group considered a full-time staffer to man the system, an RN asked “And who’s going to pay for that person? Just keeping it real.” One nephrologist repeatedly asked, “How much time is it going to take?” The dialysis clinic staff universally delegated DialysisConnect tasks to their administrative assistants, who currently handle information requests from hospitals.

Doubt that others, whether colleagues or personnel at the other site, would participate was pervasive. Participants also acknowledged that the delegation of tasks raised questions about needing to expand access to patient medical records to additional staff and instituting an escalation process if DialysisConnect tasks did not get done.

### Internalization

The ways that participants envisioned embracing DialysisConnect were limited. All groups easily imagined using DialysisConnect’s communication channel. Hospitalists, hospital staff, and nephrologists envisioned having quick exchanges with dialysis clinics about specific information requests while on the move. Dialysis clinic staff, who mentioned experience with the communication channel from a similar transplant technology, confirmed that its use was easy. All groups further embraced using DialysisConnect for patients moving between settings outside of their healthcare system for which there is no current workaround. These participants struggled to imagine uploading and downloading information not only in terms of making time to do so but also the logistical aspects of cutting and pasting or attaching within their respective EMRs. They looked to other electronic systems they had used or heard about to try to internalize it. Several could imagine instead and wanted a health information exchange or passive dashboard.

The dialysis clinic staff group alone elaborated on the value of DialysisConnect for continuity of care. They embraced the opportunity to exchange information about dialysis patients in a uniquely uncomplicated way. They described current time and effort spent to document reasons for missed dialysis, as required by CMS CROWNWeb, a data management system used by most U.S. dialysis facilities to submit required facility and patient data to CMS, including doing welfare checks at patients’ homes. They reported other waste in the absence of timely hospitalization information including duplicating services already ordered by hospitalists. In addition to having more information, dialysis clinic staff also embraced the opportunity to communicate, when possible, with hospital entry points such as emergency departments about their patients’ diagnoses and patient history. They described sequelae of not having this communication, such as searching for absent patients who might be hospitalized, inability to continue care prescribed by hospitalists, or medical errors due to lack of information. Their additional concerns included double ordering of services, antibiotic resistance, and readmissions. The addition of DialysisConnect would mean, according to one social worker, simply “giving more continuity of care.”

## Discussion

Our thematic analysis of the perceived coherence of DialysisConnect to potential users highlights the complications of implementing new technologies into clinical settings. The stakeholders at the hospital and dialysis clinics, which are affiliated with a single healthcare system but not on the same EHR, clearly identified problems with communication between the two settings, including lack of baseline information on dialysis patients admitted to the hospital and lack of hospital discharge information such as antibiotics and services ordered when patients return to their dialysis clinic. However, after viewing the DialysisConnect simulation, all stakeholder groups except the dialysis clinic staff weighed perceived effort against benefit and more readily embraced current workarounds than the new technology. Even without interoperability of EHRs, only when considering the technology in the context of outside health systems without joint privileges and health information exchanges did they see a clear benefit and coherence for DialysisConnect. In this case, the health system’s exceptional resources were a deterrent to the coherence of DialysisConnect.

Communal specification, in terms of perceived effort to benefit in the immediate context, as well as individual specification in terms of perceived personal responsibilities and tasks, tipped DialysisConnect towards incoherence. This was most clear where a consensus was that responsibility for manning the system at discharge would fall to a single social worker, who then questioned the need for such a system and suspected dialysis clinics of exaggerating the problem. Unclear efficiencies within the system also blocked the ability of some participants to favorably differentiate DialysisConnect from the current system with all its flaws.

The importance of NPT’s coherence construct to subsequent staff buy-in and participation has been demonstrated in long-term care [17], hospital perioperative care [18, 19], and primary care [20]. A process evaluation of an enhanced-recovery-after-surgery program found that implementation champions’ sense-making was critical to uptake [18, 19]. Divergent understandings among healthcare team members of the principles behind a shared decision making program worsened as the implementation progressed [20]. Many of these evaluations, like ours, were conducted at the pre-implementation stage. We found value in measuring the construct of coherence at the outset of the project to identify anticipated issues with the system that we could address in advance of launch. We understand from speaking with the hospitalists and hospital staff in particular that DialysisConnect will need to provide as much information as possible by expanding automated (vs. manual) input from dialysis clinics and allowing system initiation from the dialysis clinic (vs. just hospital) side to obviate the need to request documents from dialysis clinics. Nephrologists and hospitalists also noted that requiring patient initiation into the system on the hospital side, as originally designed, could prevent dialysis clinics from getting discharge information if this did not occur. Initiation on either the dialysis or the hospital side is a future planned change.

Our findings identify ways that DialysisConnect should be messaged to enhance coherence for potential users. It will be important to make providers aware that DialysisConnect is being piloted for transitions of care in the typical healthcare system in which dialysis clinics and hospitals are not jointly owned. Indeed, about 80% of U.S. dialysis clinics are owned by two large dialysis organizations, Davita and Fresenius [22], both for-profit companies with their own proprietary EHRs. Providers may also need reminding of CMS penalties for hospital readmissions; none of the focus group participants mentioned them, and only dialysis clinic staff implicated poor communication between hospitals and dialysis clinics in these readmissions. It is important to emphasize to potential users that the responsibility shared across dialysis clinics and hospitals in reducing these readmissions, as well as potential solutions such as DialysisConnect, are a communal enterprise.

Our findings further provided information about how stakeholders misunderstand processes at the other healthcare setting and represent an opportunity to clarify those processes to raise awareness of the need for improvement. For example, hospitalists perceived that 95% of dialysis patient hospitalizations come directly from the dialysis clinics, suggesting that dialysis clinics should be the main initiators and drivers of the DialysisConnect system during hospitalization. However, dialysis clinic staff, who have an ongoing relationship with these patients, estimated this number at closer to 10% and the rest as coming to the hospital from the community. This perceptual divide points to how DialysisConnect might play a role in collecting dialysis patient entry point prospectively and raising awareness about the gap in care transition information delivery. Informing hospitalists and hospital staff that dialysis clinic nephrologists and staff spend valuable time looking for missing dialysis patients, who may be variously nonadherent, hospitalized, distressed, or dead, and the need to fill out CMS CROWNWeb documentation on reason for absence, could improve coherence for those on the hospital side. Noting the experience of dialysis clinic staff with a similar technology for managing kidney transplants as “easy” could alleviate anxiety for hospital users. Finally, presenting the three ways that DialysisConnect was differentiated positively over current care, as a communication channel, a dashboard, and as expedited post-hospitalization information delivery, can help stakeholders envision using DialysisConnect. Some concerns, such as whether users will be assigned individual patients to track, can be addressed to indicate that the dashboard of patients is shared across all users for communal management.

Our study represents a pre-implementation assessment of coherence of a simulated system prototype (i.e., an idea) rather than user testing of an actual built system. We will learn more when practitioners actually use the system in the next phase of the project addressing other NPT constructs—namely, cognitive participation (building a new community of practice) and collective action (operationalization of practice). We expect that the system will not simply add to clinic tasks but replace some of them with more efficient methods; for example, instead of hospitals repeatedly phoning the dialysis clinics, they will be requesting information through DialysisConnect and creating a documented exchange of information. Although the burden of work may fall to certain professional roles and could be perceived as unfair, we anticipate that better downstream outcomes for the entire team over time will offset these burdens, and this has been incorporated as a key part of our messaging in the pilot. During the current pilot of this system we will also be able to test our hypothesis that improved communication through DialysisConnect will help reduce hospital readmissions.

The strength of this study of initial coherence is that it probed hospital and dialysis clinic work cultures. Some examinations of NPT constructs include only one stakeholder viewpoint [13]; we included four. However, the research was limited to one non-profit academic health system that is relatively unusual in having affiliated but separately managed hospitals and dialysis clinics. Our health system is not representative of the typical health setting where dialysis clinics are owned by for-profit companies interfacing with separately managed hospital systems. In addition, our study sample represented a subset of 198 potential stakeholders across the healthcare system, with associated potential lack of generalizability. We also note that we left out other potential system stakeholders including Emergency Department staff.

## Conclusion

Our thematic analysis of the perceived coherence of DialysisConnect to potential users highlights the complications of implementing new technologies into clinical settings and suggests ways to mitigate them. In the absence of EHR interoperability, intermediary solutions like DialysisConnect are necessary to allow basic continuity of care between dialysis clinics and hospitals. The biggest block to coherence may be a lack of hospital awareness about the information needs of dialysis clinics in the ongoing care of their dialysis patients, with whom they have a personal ongoing connection, to prevent hospitalization. We see the normalization of DialysisConnect into workflow as requiring a shift from individual to communal responsibility both within and across health settings. A composite picture from these stakeholder discussions of how the patient moves between the settings in the absence of information may improve engagement in each setting.

## Supplementary Information

**Additional file 1**. DialysisConnect prototype: Slides extracted from video shown to focus group participants with private information redacted

**Additional file 2: Table 1**. Consolidated criteria for reporting qualitative research 32-item checklist for DialysisConnect coherence study

## Data Availability

Available upon reasonable request.

## Abbreviations

ESRD: End-stage renal disease
CMS: Centers for Medicare & Medicaid Services
EHR: Electronic health record
API: Application programming interface
HIE: Healthcare information exchange
TREX: Transplant Referral Exchange
NPT: Normalization Process Theory
