# Supplementary material for: Making sense of DialysisConnect: a qualitative analysis of stakeholder viewpoints on a web-based information exchange platform to improve care transitions between dialysis clinics and hospitals
Source: BMC Med Inform Decis Mak. 2021 Feb 9;21:47. doi: 10.1186/s12911-021-01415-y (PMC7871569; doi:10.1186/s12911-021-01415-y)
Supplement: Supplementary file 1 — Additional file 1. DialysisConnect prototype: Slides extracted from video shown to focus group participants with private information redacted [file 12911_2021_1415_MOESM1_ESM.pdf]

Introduction to the DialysisConnect Application

- Hospital Admission
  - Search for Patient Information
  - Request supporting documents from Dialysis Center
- Dialysis Center – Respond to Request
  - Identify request
  - Upload requested information
- Communication Channel
- Hospital Discharge Process
- General Reporting Capabilities

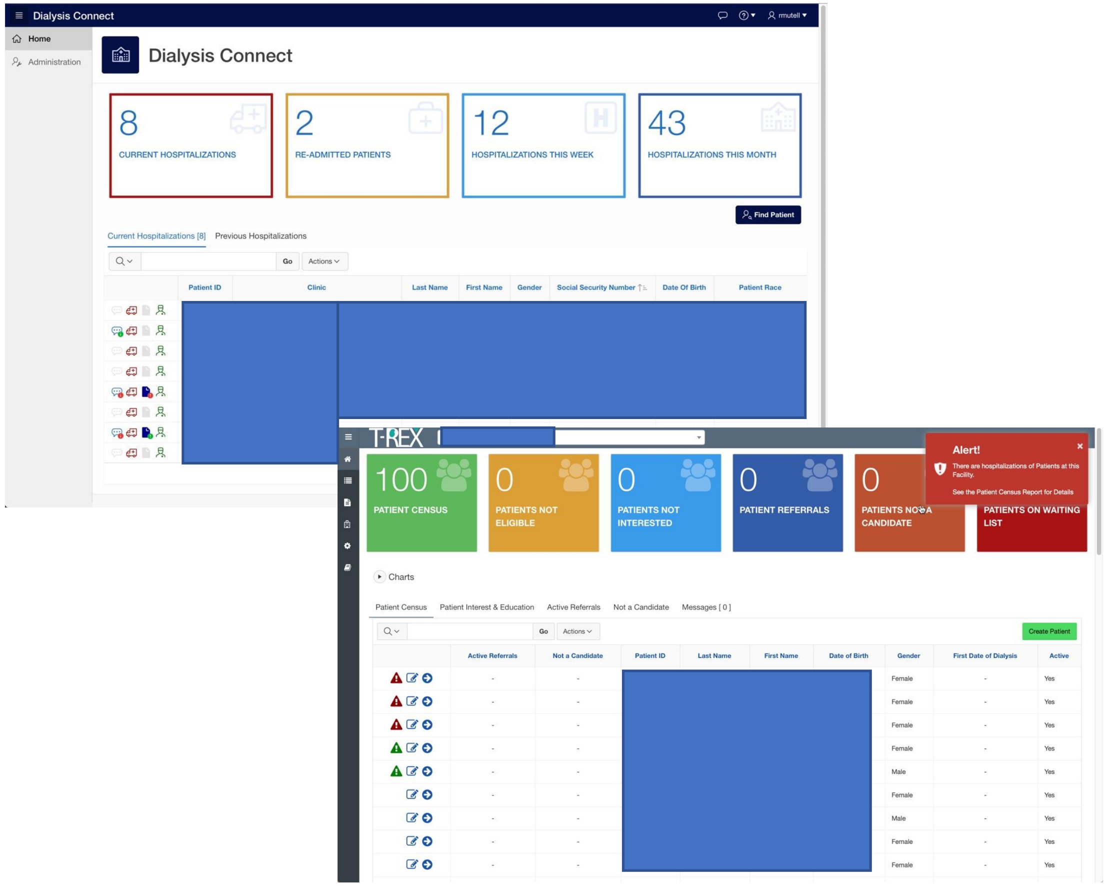

Hospital versus Dialysis View

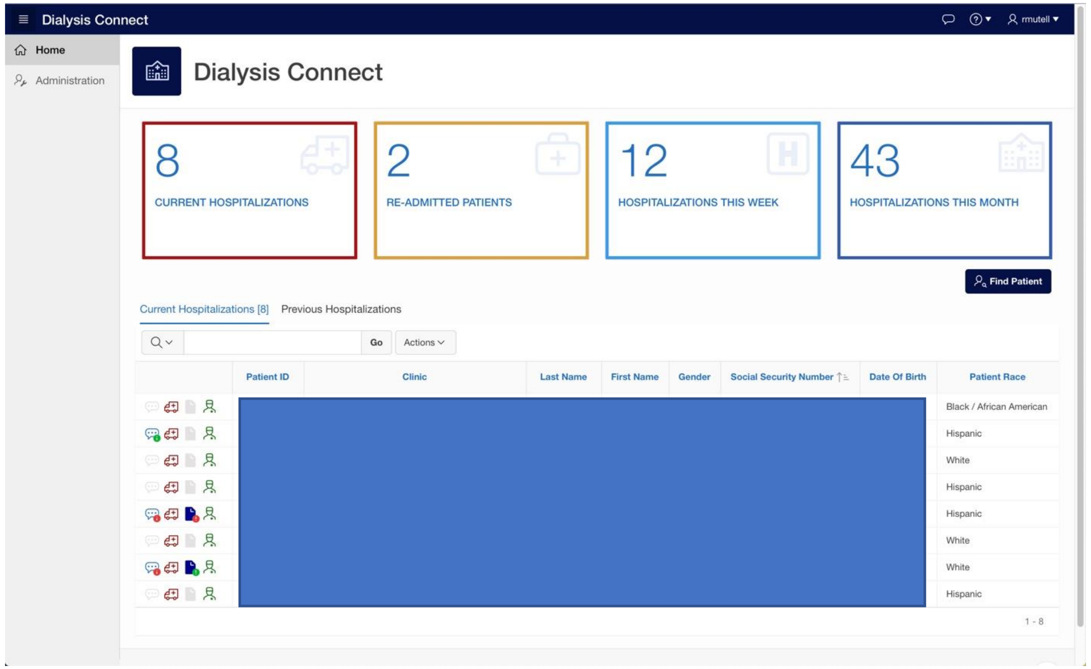

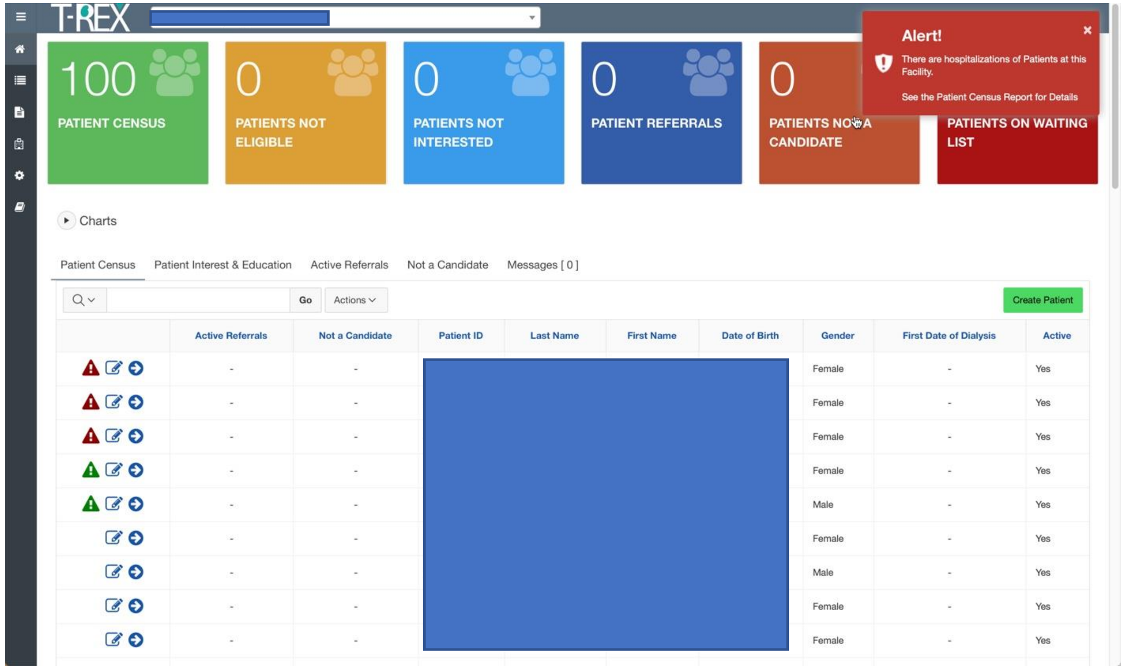

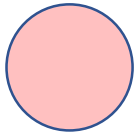

This icon will indicate where I am clicking on the screen

Patient Admitted

Find Patient
Request Information

Dialysis Connect

rmutell

••••••••

Remember username

Sign In

To reset your password click this link
To request access to Dialysis Connect click this link

Dialysis Connect

rmutell

Home

Administration

Dialysis Connect

8
CURRENT HOSPITALIZATIONS

2
RE-ADMITTED PATIENTS

12
HOSPITALIZATIONS THIS WEEK

43
HOSPITALIZATIONS THIS MONTH

Find Patient

Current Hospitalizations [8] | Previous Hospitalizations

Go | Actions

	Patient ID	Clinic	Last Name	First Name	Gender	Social Security Number	Date Of Birth	Patient Race
	[redacted]							Black / African American
								Hispanic
								White
								Hispanic
								Hispanic
								White
								White
								Hispanic

1 - 8

Dialysis Connect

rmutell

Dialy

8

CURRENT HOSPI

THIS MONTH

Find Patient

Current Hospitalizatio

Find Patient

×

Patient Quick Search

Advanced Search

Patient Race
Black / African American
Hispanic
White
Hispanic
Hispanic
White
White
Hispanic

1 - 8

Dialysis Connect

rmutell

Dial

8

CURRENT HOSP

THIS MONTH

Find Patient

Current Hospitalizatio

Find Patient

Patient Quick Search

Patient Race
Black / African American
Hispanic
White
Hispanic
Hispanic
White
White
Hispanic

1 - 8

Dialysis Connect

rmutell

Dialys

8

CURRENT HOSPITA

IS THIS MONTH

Find Patient

Current Hospitalizations

Patient Race
Black / African American
Hispanic
White
Hispanic
Hispanic
White
White
Hispanic

1 - 8

Patient Hospitalization

[redacted]

Patient Demographics | Nephrologist Information | Clinic | Medical Information | Emergency Contact

[redacted]

Home Phone

Cell Phone

Email Address

Initial Hospitalization Reasons

Include	Reason
☐	Hospitalization Reason 1
☐	Hospitalization Reason 2
☐	Hospitalization Reason 3
☐	Hospitalization Reason 4

Cancel

Request

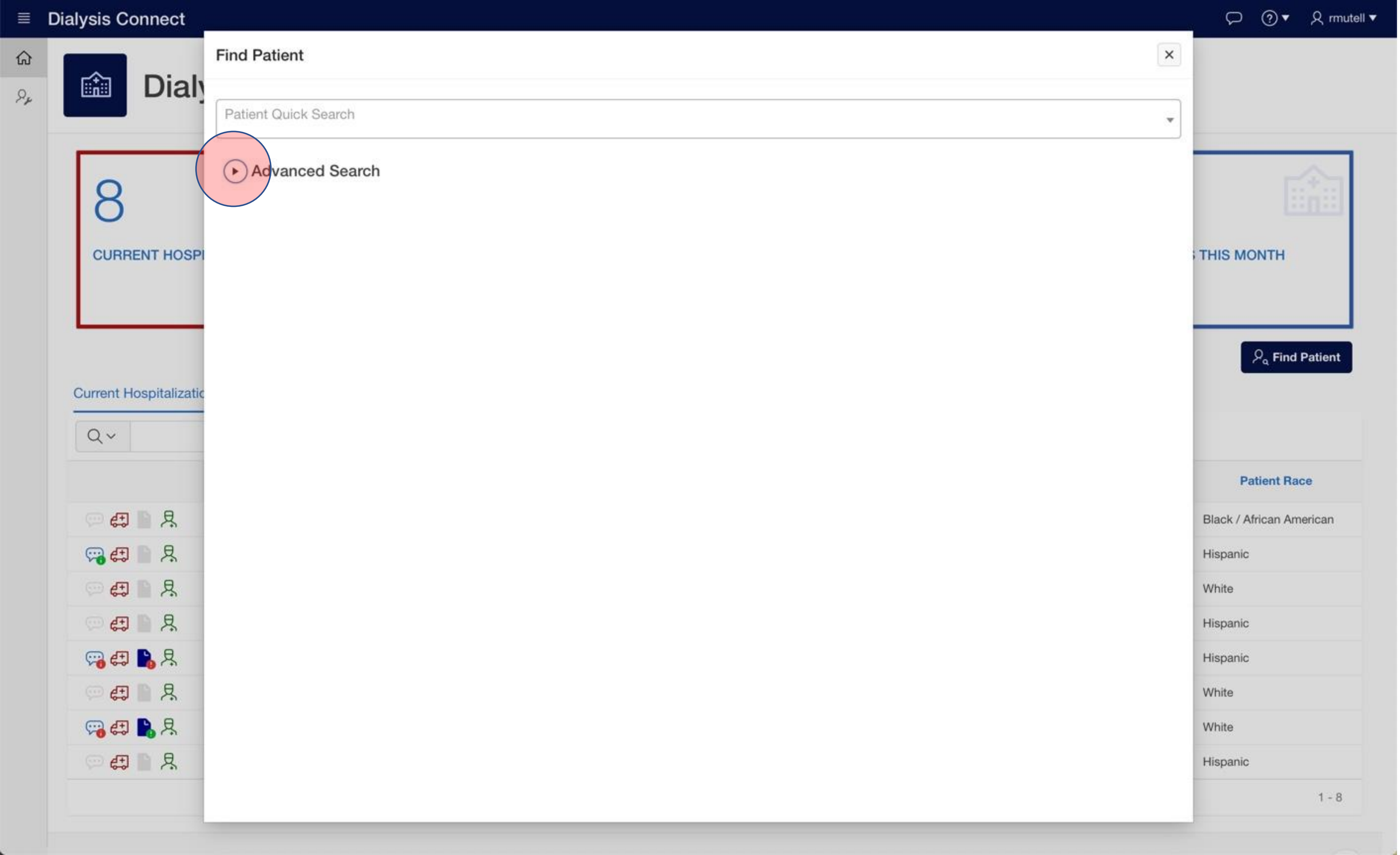
Dialysis Connect
rmutell
Find Patient
Patient Quick Search
Advanced Search
8
CURRENT HOSP
THIS MONTH
Find Patient
Current Hospitalizatio
Patient Race
Black / African American
Hispanic
White
Hispanic
Hispanic
White
White
Hispanic
1 - 8

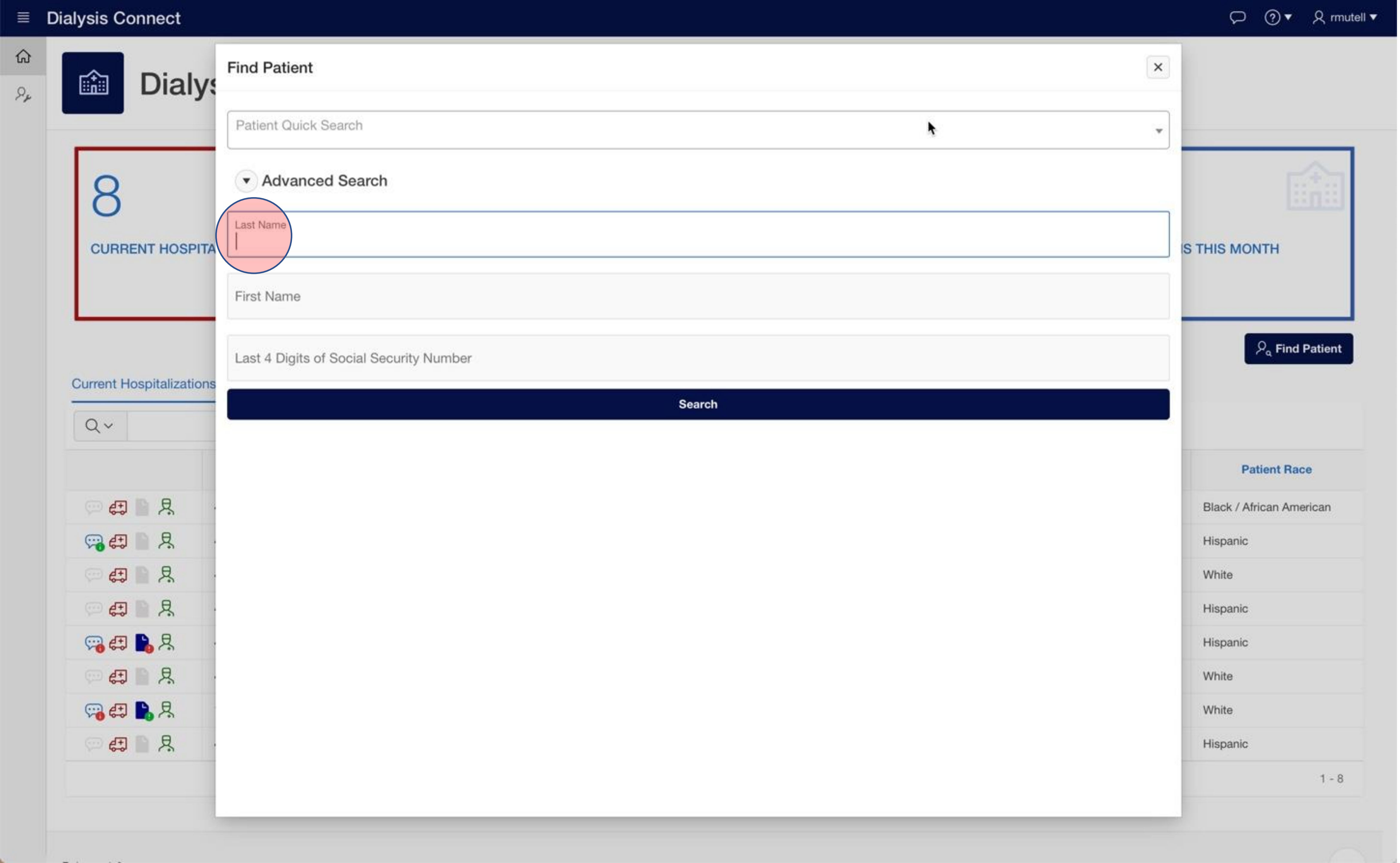
Dialysis Connect
rmutell
Find Patient
Patient Quick Search
Advanced Search
Last Name
First Name
Last 4 Digits of Social Security Number
Search
8
Find Patient
Current Hospitalizations
Patient Race
Black / African American
Hispanic
White
Hispanic
Hispanic
White
White
Hispanic
1 - 8

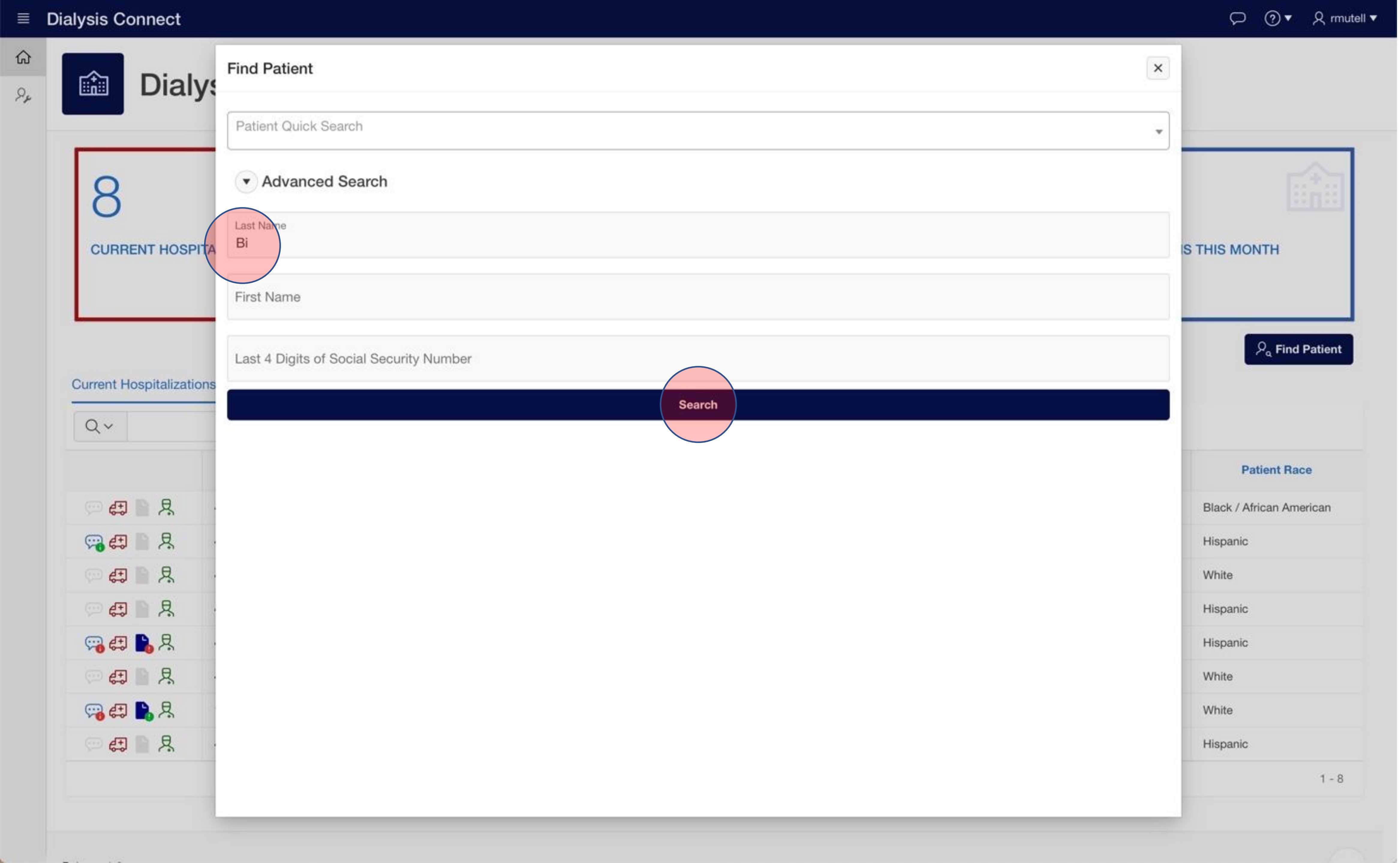
Dialysis Connect
rmutell
Dialys
8
CURRENT HOSPITA
S THIS MONTH
Find Patient
Current Hospitalizations
Patient Race
Black / African American
Hispanic
White
Hispanic
Hispanic
White
White
Hispanic
1 - 8
Find Patient
Patient Quick Search
Advanced Search
Last Name
Bi
First Name
Last 4 Digits of Social Security Number
Search

Dialysis Connect

rmutell

Dialys

8

CURRENT HOSPITA

S THIS MONTH

Find Patient

Current Hospitalizations

Find Patient

Patient Quick Search

Advanced Search

Link	Patient ID	Facility	First Name	Last Name	Gender	Date Of Birth	Patient Race	Social Security Number
	[redacted]							

1 - 3

Patient Race
Black / African American
Hispanic
White
Hispanic
Hispanic
White
White
Hispanic

1 - 8

Dialysis Connect rmutell

Dial

8

CURRENT HOSP

Current Hospitalizatio

THIS MONTH

Find Patient

Patient Hospitalization

[redacted]

Patient Demographics | Nephrologist Information | Clinic | Medical Information | Emergency Contact

Social Security Number	Date of Birth	Age
[redacted]		

Other Language	Gender	Race
	Female	Black / African American

Street Address	City	State	Zip Code
[redacted]			

Home Phone	Cell Phone	Email Address

Initial Hospitalization Reasons

Include	Reason
☐	Hospitalization Reason 1
☐	Hospitalization Reason 2
☐	Hospitalization Reason 3
☐	Hospitalization Reason 4

Cancel

Request

Patient Race
Black / African American
Hispanic
White
Hispanic
Hispanic
White
White
Hispanic

1 - 8

Dialysis Connect

rmutell

Dial

8

CURRENT HOSP

THIS MONTH

Find Patient

Current Hospitalizatio

Patient Hospitalization

[redacted]

Patient Demographics | Nephrologist Information | Clinic | Medical Information | Emergency Contact

Referral Date

[redacted]

Nephrologist Name

Nephrologist Phone Number

Physician Fax

Email

Initial Hospitalization Reasons

Include	Reason
☐	Hospitalization Reason 1
☐	Hospitalization Reason 2
☐	Hospitalization Reason 3
☐	Hospitalization Reason 4

Cancel

Request

Patient Race
Black / African American
Hispanic
White
Hispanic
Hispanic
White
White
Hispanic

1 - 8

Dialysis Connect rmutell

Dialy

8

CURRENT HOSPI

Current Hospitalizatio

Patient Hospitalization

[redacted]

Patient Demographics | Nephrologist Information | Clinic | Medical Information | Emergency Contact

Dialysis Schedule
M-W-F

Dialysis Center

Provider ID

Dialysis Phone Number

Dialysis Street Address | Dialysis City | Dialysis State | Dialysis Zip Code

Dialysis Contact

Contact Email

Initial Hospitalization Reasons

Cancel

Request

THIS MONTH

Find Patient

Patient Race
Black / African American
Hispanic
White
Hispanic
Hispanic
White
White
Hispanic

1 - 8

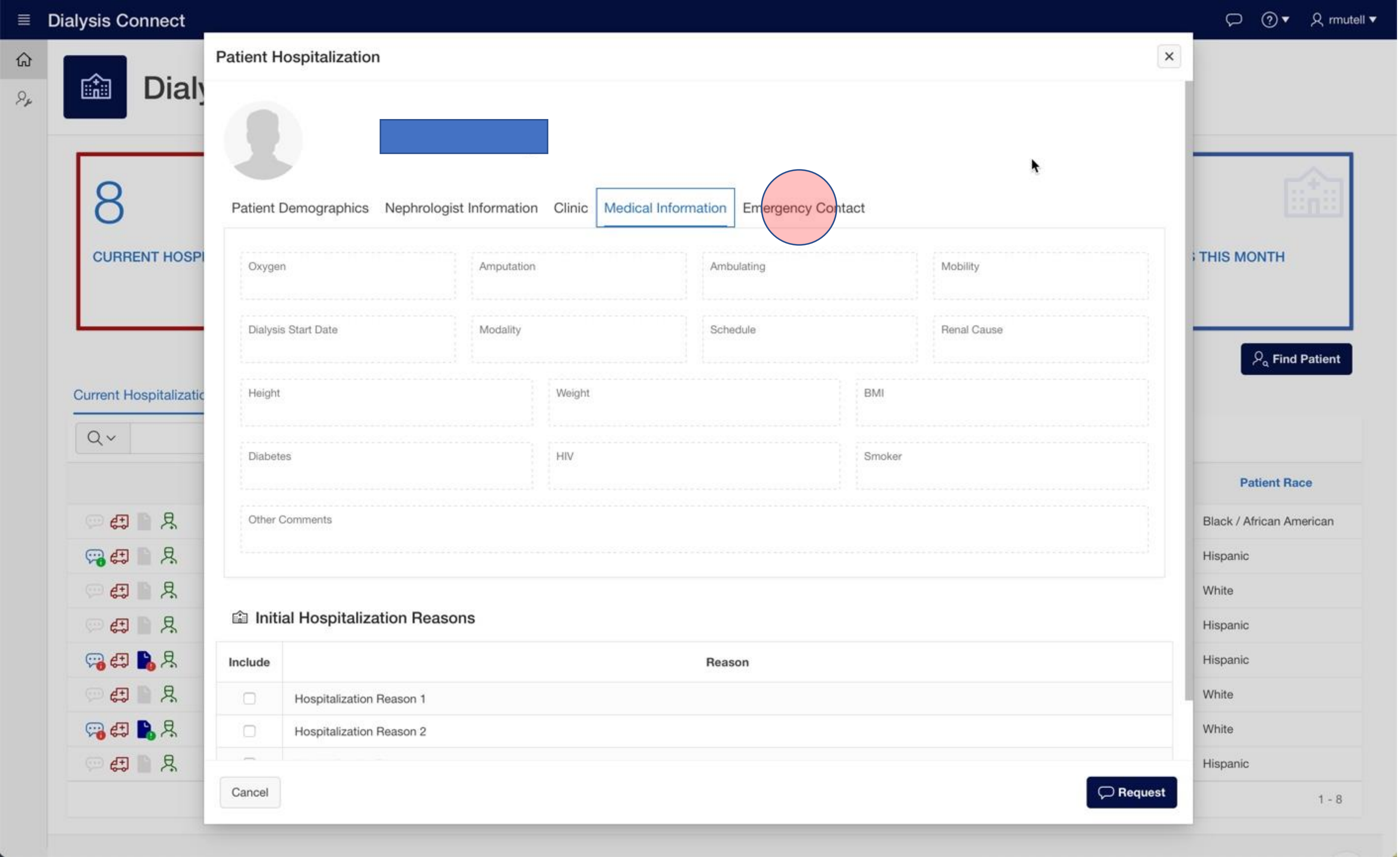
Dialysis Connect
rmutell
Dial
8
CURRENT HOSP
THIS MONTH
Find Patient
Current Hospitalizati
Patient Race
Black / African American
Hispanic
White
Hispanic
Hispanic
White
White
Hispanic
1 - 8
Patient Hospitalization
Patient Demographics
Nephrologist Information
Clinic
Medical Information
Emergency Contact
Oxygen
Amputation
Ambulating
Mobility
Dialysis Start Date
Modality
Schedule
Renal Cause
Height
Weight
BMI
Diabetes
HIV
Smoker
Other Comments
Initial Hospitalization Reasons
Include
Reason
Hospitalization Reason 1
Hospitalization Reason 2
Cancel
Request

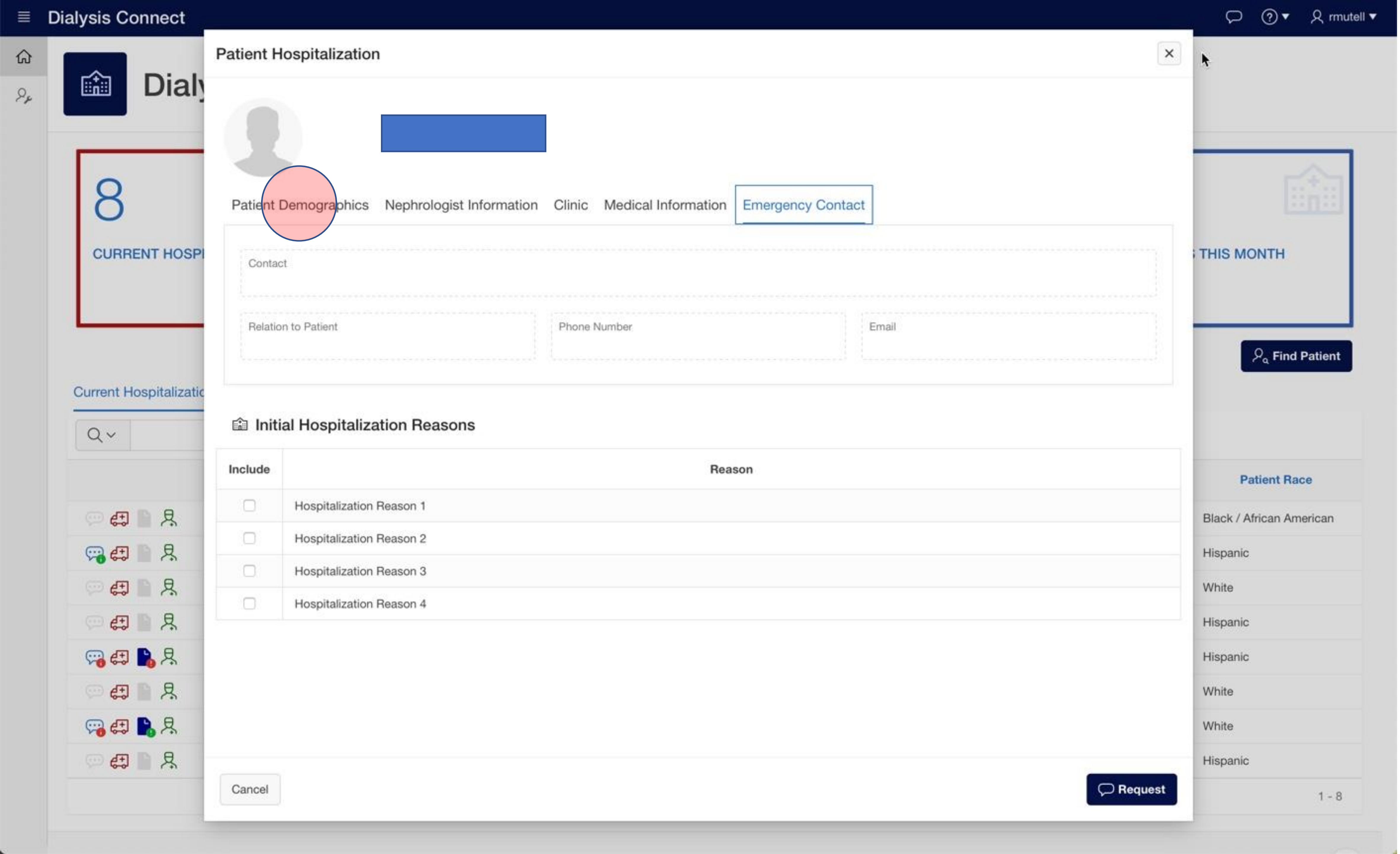
Dialysis Connect
rmutell
Patient Hospitalization
Patient Demographics
Nephrologist Information
Clinic
Medical Information
Emergency Contact
Contact
Relation to Patient
Phone Number
Email
Initial Hospitalization Reasons
Include
Reason
Hospitalization Reason 1
Hospitalization Reason 2
Hospitalization Reason 3
Hospitalization Reason 4
Cancel
Request
8
CURRENT HOSPI
THIS MONTH
Find Patient
Current Hospitalizatio
Patient Race
Black / African American
Hispanic
White
Hispanic
Hispanic
White
White
Hispanic
1 - 8

Dialysis Connect rmutell

Dialy

8

CURRENT HOSP

THIS MONTH

Find Patient

Current Hospitalizatio

Patient Hospitalization

[redacted]

Patient Demographics | Nephrologist Information | Clinic | Medical Information | Emergency Contact

Social Security Number | Date of Birth | Age

[redacted]

Other Language

Gender: Female

Race: Black / African American

Street Address | City | State | Zip Code

[redacted]

Home Phone

Cell Phone

Email Address

Initial Hospitalization Reasons

Include	Reason
☐	Hospitalization Reason 1
☐	Hospitalization Reason 2
☐	Hospitalization Reason 3
☐	Hospitalization Reason 4

Cancel Request

Patient Race
Black / African American
Hispanic
White
Hispanic
Hispanic
White
White
Hispanic

1 - 8

Dialysis Connect

rmutell

Dial

8

CURRENT HOSP

THIS MONTH

Find Patient

Current Hospitalizati

Patient Hospitalization

[redacted]

Patient Demographics | Nephrologist Information | Clinic | Medical Information | Emergency Contact

Social Security Number | Date of Birth | Age

[redacted]

Other Language

Gender: Female

Race: Black / African American

Street Address | City | State | Zip Code

[redacted]

Home Phone

Cell Phone

Email Address

Initial Hospitalization Reasons

Include	Reason
☑	Hospitalization Reason 1
☑	Hospitalization Reason 2
☐	Hospitalization Reason 3
☐	Hospitalization Reason 4

Cancel

Request

Patient Race
Black / African American
Hispanic
White
Hispanic
Hispanic
White
White
Hispanic

1 - 8

Dialysis Connect

Request for information has been sent to the dialysis clinic

Dialysis Connect

8
CURRENT HOSPITALIZATIONS

2
RE-ADMITTED PATIENTS

12
HOSPITALIZATIONS THIS WEEK

43
HOSPITALIZATIONS THIS MONTH

Find Patient

Current Hospitalizations [8] | Previous Hospitalizations

Go | Actions

	Patient ID	Clinic	Last Name	First Name	Gender	Social Security Number	Date Of Birth	Patient Race
	[redacted]							Black / African American
	[redacted]							Hispanic
	[redacted]							White
	[redacted]							Hispanic
	[redacted]							Hispanic
	[redacted]							White
	[redacted]							White
	[redacted]							Hispanic

1 - 8

Log into Dialysis Application

Respond to Request

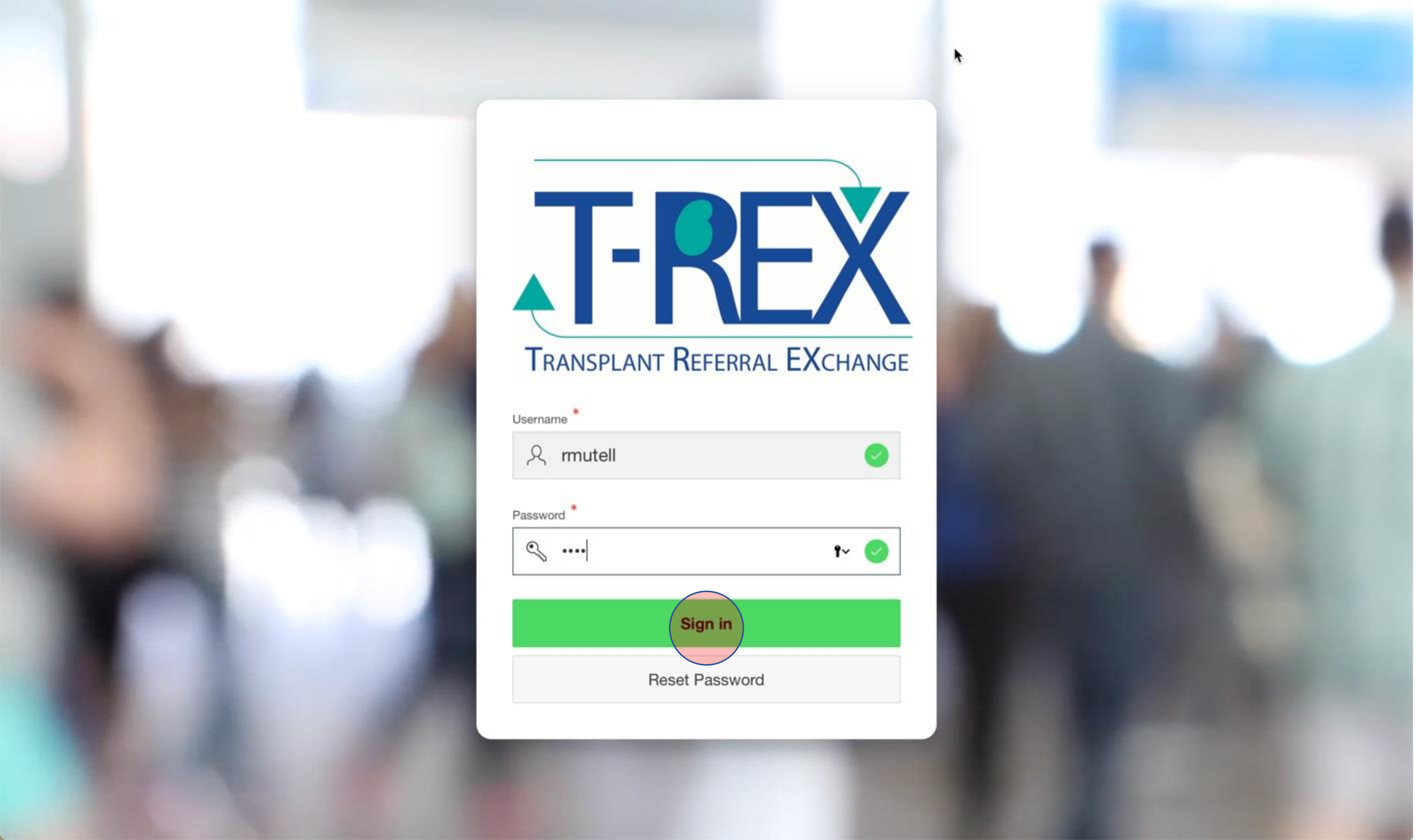
T-REX
TRANSPLANT REFERRAL EXCHANGE
Username *
rmutell
Password *
Sign in
Reset Password

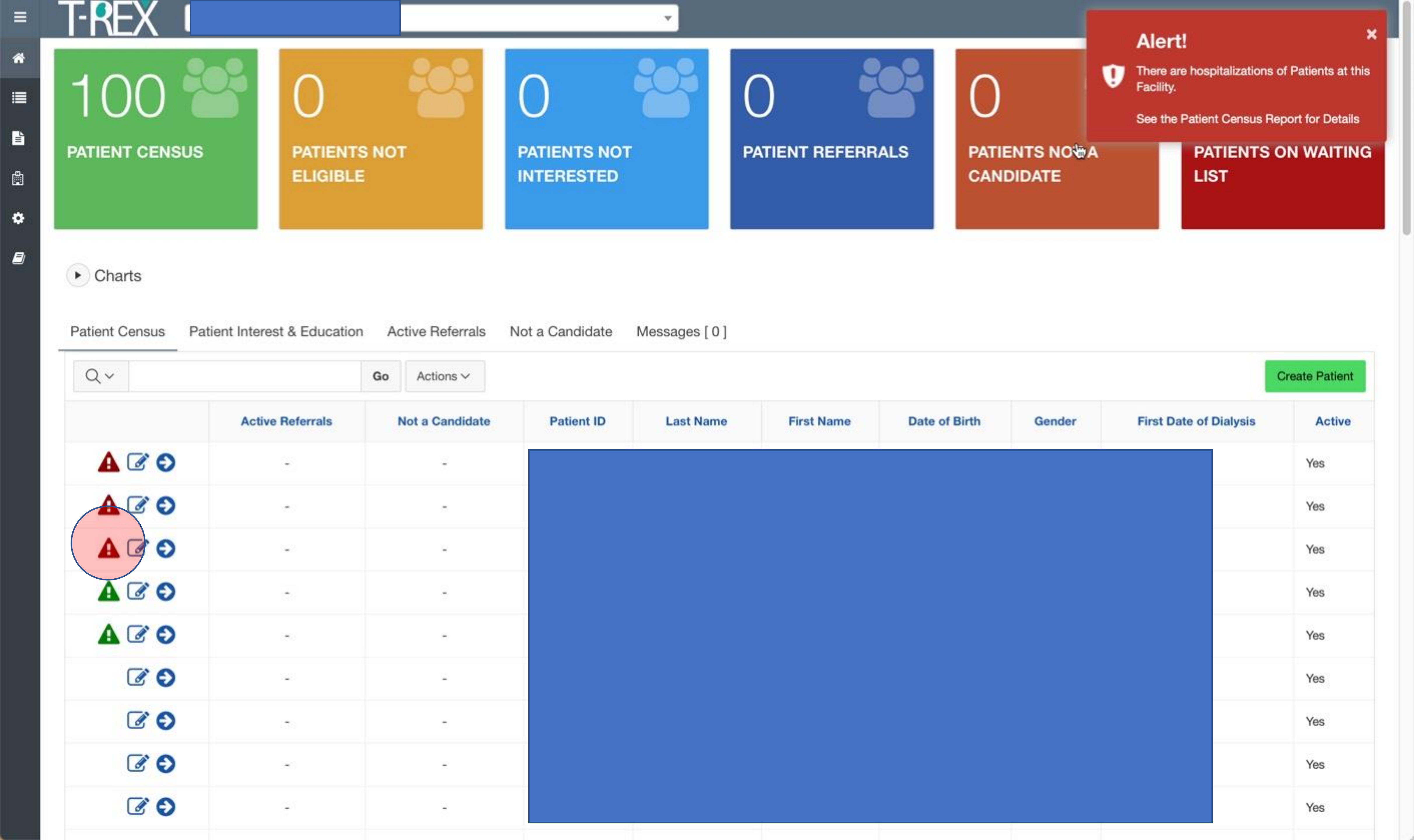
T-REX
100
PATIENT CENSUS
0
PATIENTS NOT ELIGIBLE
0
PATIENTS NOT INTERESTED
0
PATIENT REFERRALS
0
PATIENTS NOT A CANDIDATE
PATIENTS ON WAITING LIST
Alert!
There are hospitalizations of Patients at this Facility.
See the Patient Census Report for Details
Charts
Patient Census
Patient Interest & Education
Active Referrals
Not a Candidate
Messages [0]
Go
Actions
Create Patient
Active Referrals
Not a Candidate
Patient ID
Last Name
First Name
Date of Birth
Gender
First Date of Dialysis
Active
Yes
Yes
Yes
Yes
Yes
Yes
Yes
Yes
Yes

T-REX

Feedback

100

PATIENT CENSUS

ATIENTS ON WAITING
ST

Charts

Patient Census | Patie

Create Patient

	Dialysis	Active
		Yes
		Yes
		Yes
		Yes
		Yes
		Yes
		Yes
		Yes
		Yes

Upload Documents

×

Required Documents

Reason	Required
Requested Information 1	Yes
Requested Information 2	Yes
Requested Information 3	Yes
Requested Information 4	Yes
Requested Information 5	Yes
Requested Information 6	Yes
Requested Information 7	Yes
Requested Information 8	Yes

Download

1 - 8

Upload Documents

Drop files here or click to upload.

Cancel

Send to Hospital

Save

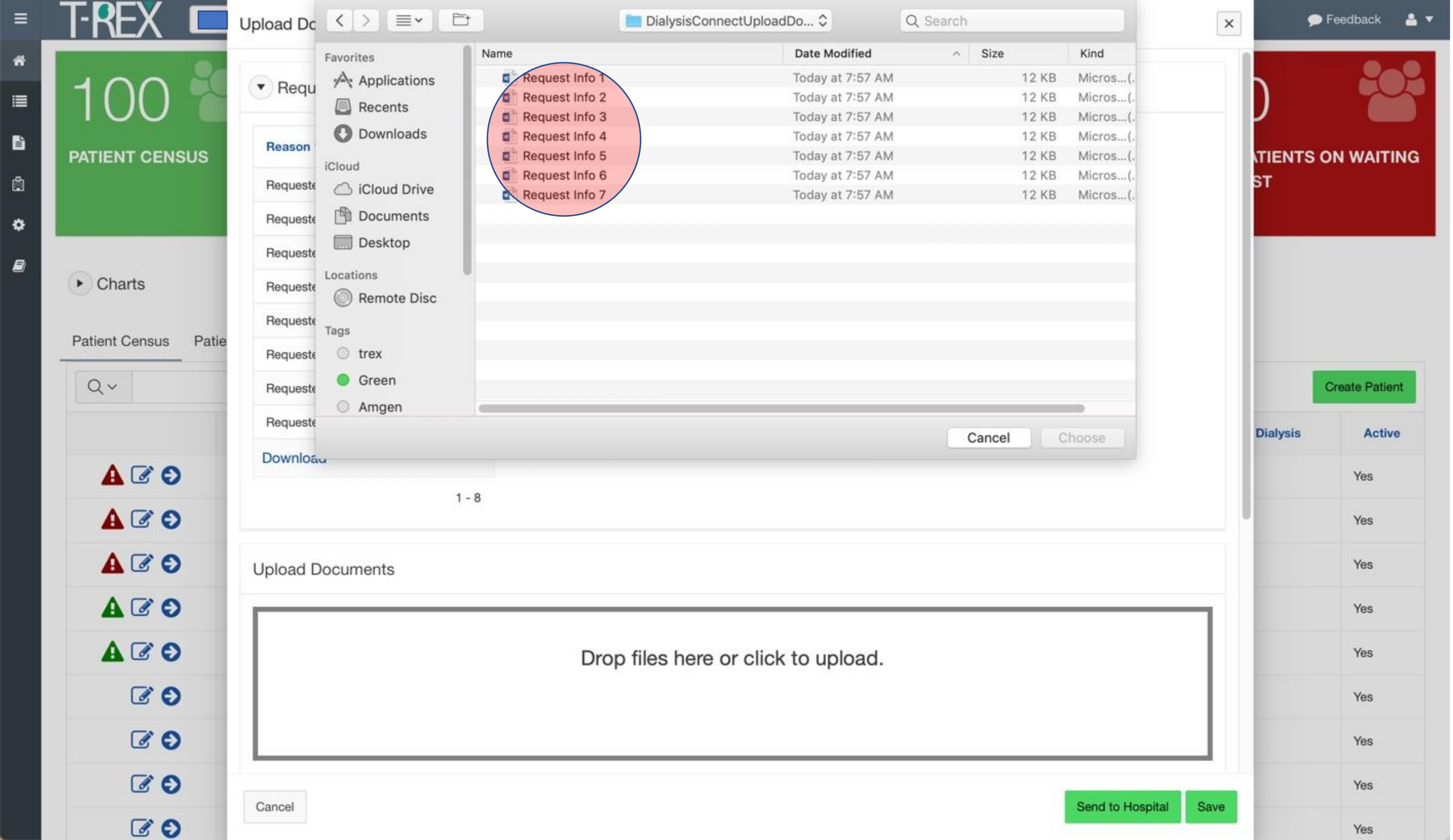
T-REX
Upload Dc
DialysisConnectUploadDo...
Search
Feedback
Favorites
Applications
Recents
Downloads
iCloud
iCloud Drive
Documents
Desktop
Locations
Remote Disc
Tags
trex
Green
Amgen
Name
Date Modified
Size
Kind
Request Info 1 Today at 7:57 AM 12 KB Micros...
Request Info 2 Today at 7:57 AM 12 KB Micros...
Request Info 3 Today at 7:57 AM 12 KB Micros...
Request Info 4 Today at 7:57 AM 12 KB Micros...
Request Info 5 Today at 7:57 AM 12 KB Micros...
Request Info 6 Today at 7:57 AM 12 KB Micros...
Request Info 7 Today at 7:57 AM 12 KB Micros...
Cancel
Choose
100
PATIENT CENSUS
ATIENTS ON WAITING
ST
Requ
Reason
Charts
Patient Census
Patie
Create Patient
Dialysis
Active
Yes
1 - 8
Upload Documents
Drop files here or click to upload.
Cancel
Send to Hospital
Save

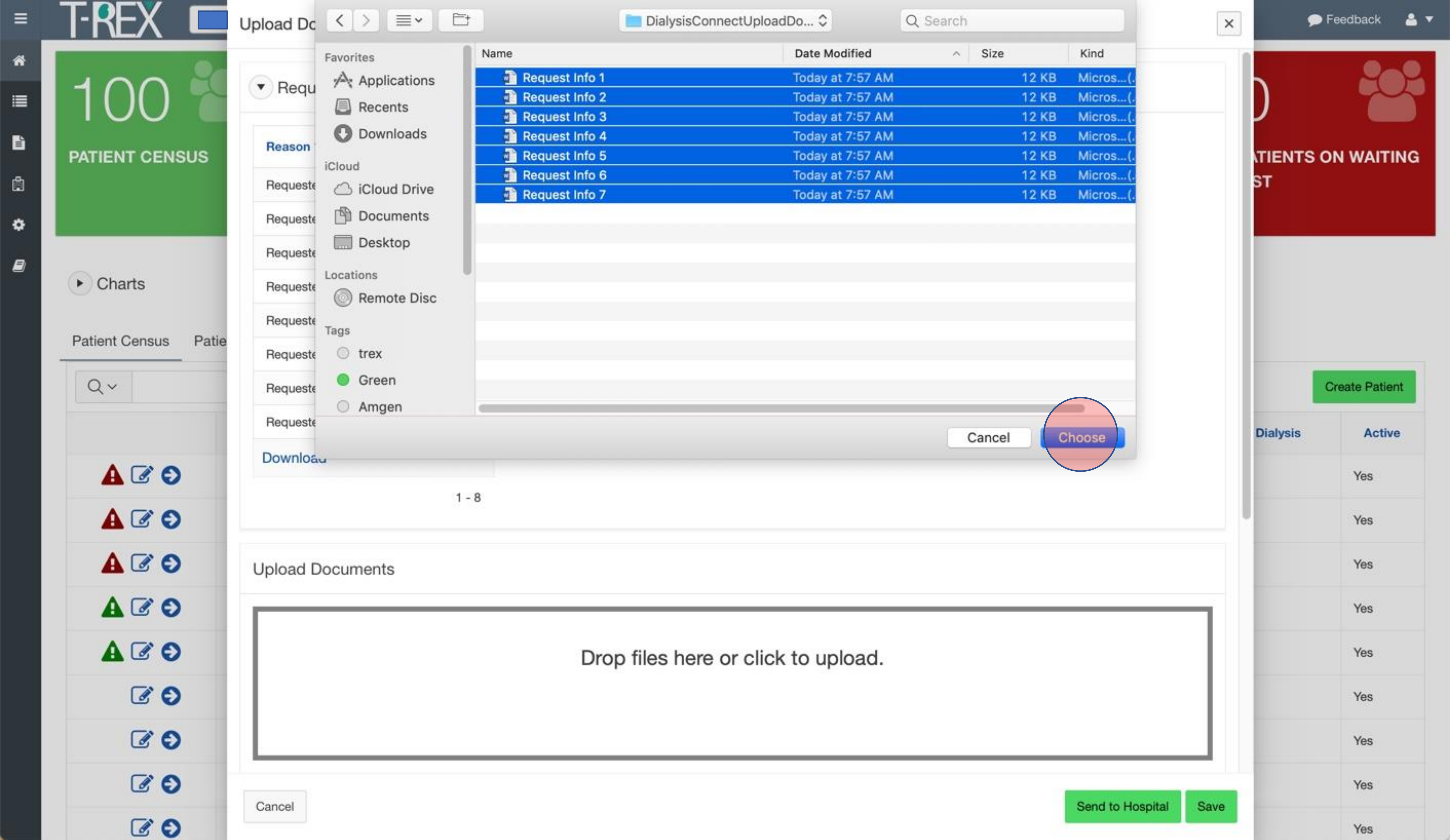
T-REX
Upload Dc
DialysisConnectUploadDo...
Search
Feedback
Favorites
Applications
Recents
Downloads
iCloud
iCloud Drive
Documents
Desktop
Locations
Remote Disc
Tags
trex
Green
Amgen
Name
Date Modified
Size
Kind
Request Info 1 Today at 7:57 AM 12 KB Micros...(.
Request Info 2 Today at 7:57 AM 12 KB Micros...(.
Request Info 3 Today at 7:57 AM 12 KB Micros...(.
Request Info 4 Today at 7:57 AM 12 KB Micros...(.
Request Info 5 Today at 7:57 AM 12 KB Micros...(.
Request Info 6 Today at 7:57 AM 12 KB Micros...(.
Request Info 7 Today at 7:57 AM 12 KB Micros...(.
Cancel
Choose
100
PATIENT CENSUS
ATIENTS ON WAITING
ST
Requ
Reason
Requeste
Charts
Patient Census
Patie
Create Patient
Dialysis
Active
Yes
1 - 8
Upload Documents
Drop files here or click to upload.
Cancel
Send to Hospital
Save

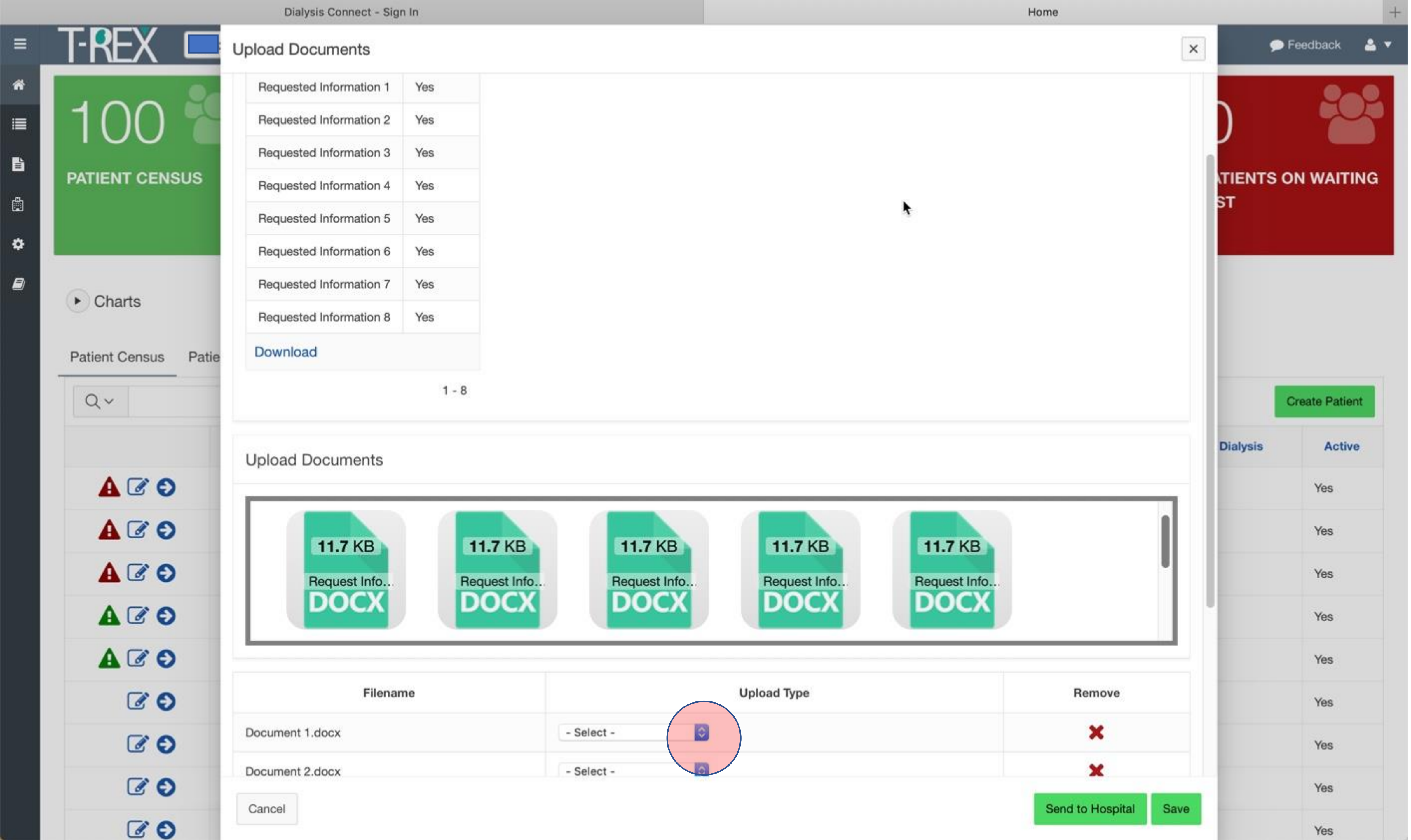
Dialysis Connect - Sign In
Home
T-REX
Upload Documents
Feedback
100
PATIENT CENSUS
ATIENTS ON WAITING
ST
Requested Information 1 Yes
Requested Information 2 Yes
Requested Information 3 Yes
Requested Information 4 Yes
Requested Information 5 Yes
Requested Information 6 Yes
Requested Information 7 Yes
Requested Information 8 Yes
Download
1 - 8
Charts
Patient Census
Patie
Create Patient
Dialysis
Active
Upload Documents
11.7 KB
Request Info...
DOCX
Filename
Upload Type
Remove
Document 1.docx
- Select -
Document 2.docx
- Select -
Cancel
Send to Hospital
Save
Yes

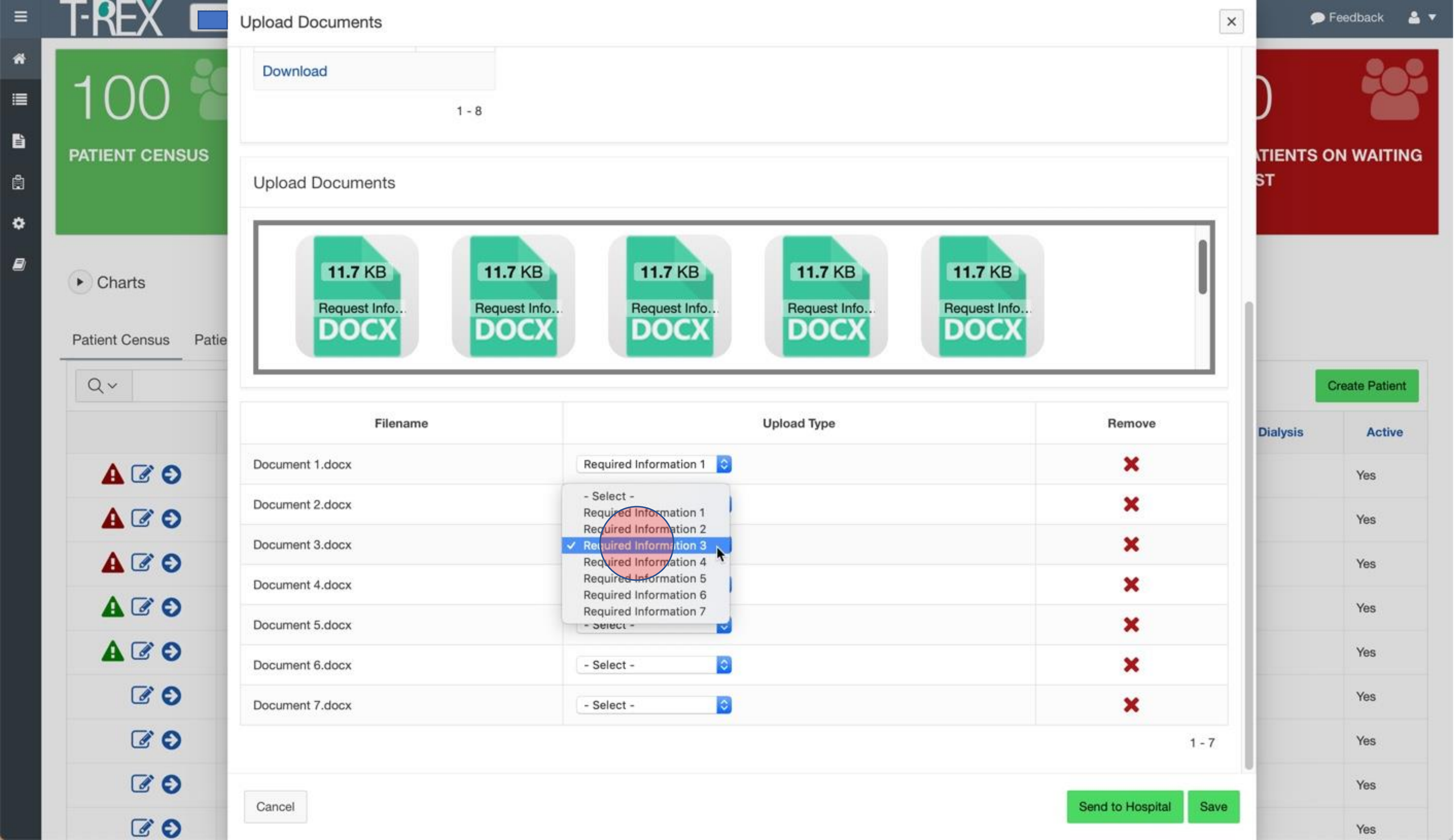
T-REX
Feedback
100
PATIENT CENSUS
ATIENTS ON WAITING
ST
Charts
Patient Census
Patie
Create Patient
Dialysis
Active
Upload Documents
Download
1 - 8
Upload Documents
11.7 KB
Request Info...
DOCX
Filename
Upload Type
Remove
Document 1.docx
Required Information 1
Document 2.docx
- Select -
Required Information 1
Required Information 2
Required Information 3
Required Information 4
Required Information 5
Required Information 6
Required Information 7
Document 3.docx
Document 4.docx
Document 5.docx
Document 6.docx
- Select -
Document 7.docx
- Select -
1 - 7
Yes
Cancel
Send to Hospital
Save

T-REX

Feedback

100

PATIENT CENSUS

ATIENTS ON WAITING
ST

Charts

Patient Census | Patie

Create Patient

	Dialysis	Active
		Yes
		Yes
		Yes
		Yes
		Yes
		Yes
		Yes
		Yes
		Yes

Upload Documents

Download

1 - 8

Upload Documents

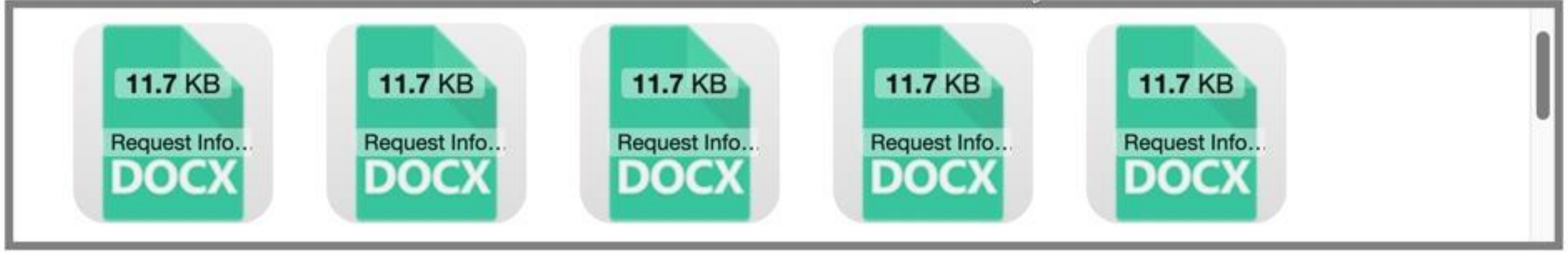

Filename	Upload Type	Remove
Document 1.docx	Required Information 1	✖
Document 2.docx	Required Information 2	✖
Document 3.docx	Required Information 3	✖
Document 4.docx	Required Information 4	✖
Document 5.docx	Required Information 5	✖
Document 6.docx	Required Information 6	✖
Document 7.docx	Required Information 7	✖

1 - 7

Cancel | Send to Hospital | Save

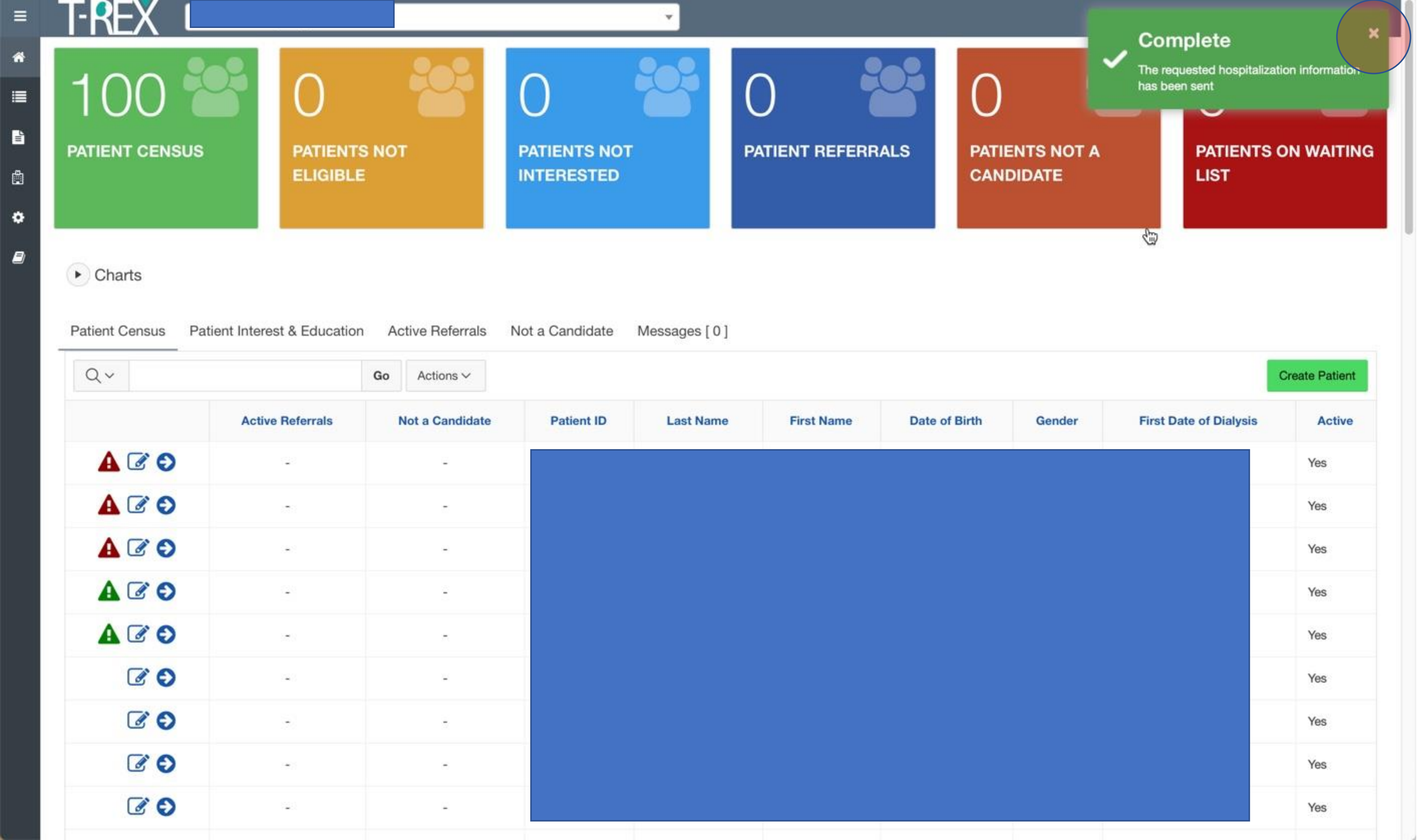
T-REX
Complete
The requested hospitalization information has been sent
100
PATIENT CENSUS
0
PATIENTS NOT ELIGIBLE
0
PATIENTS NOT INTERESTED
0
PATIENT REFERRALS
0
PATIENTS NOT A CANDIDATE
PATIENTS ON WAITING LIST
Charts
Patient Census
Patient Interest & Education
Active Referrals
Not a Candidate
Messages [0]
Go
Actions
Create Patient
Active Referrals
Not a Candidate
Patient ID
Last Name
First Name
Date of Birth
Gender
First Date of Dialysis
Active
Yes
Yes
Yes
Yes
Yes
Yes
Yes
Yes
Yes

Back to Hospital

Dialysis Connect

You have new documents from Emory Dialysis - Greenbria

Dialysis Connect

8	2	12	43
CURRENT HOSPITALIZATIONS	RE-ADMITTED PATIENTS	HOSPITALIZATIONS THIS WEEK	HOSPITALIZATIONS THIS MONTH

Find Patient

Current Hospitalizations [8] | Previous Hospitalizations

Go | Actions

Patient ID	Clinic	Last Name	First Name	Gender	Social Security Number	Date Of Birth	Patient Race
[redacted]	[redacted]	[redacted]	[redacted]	[redacted]	[redacted]	[redacted]	Black / African American
[redacted]	[redacted]	[redacted]	[redacted]	[redacted]	[redacted]	[redacted]	Hispanic
[redacted]	[redacted]	[redacted]	[redacted]	[redacted]	[redacted]	[redacted]	White
[redacted]	[redacted]	[redacted]	[redacted]	[redacted]	[redacted]	[redacted]	Hispanic
[redacted]	[redacted]	[redacted]	[redacted]	[redacted]	[redacted]	[redacted]	Hispanic
[redacted]	[redacted]	[redacted]	[redacted]	[redacted]	[redacted]	[redacted]	White
[redacted]	[redacted]	[redacted]	[redacted]	[redacted]	[redacted]	[redacted]	White
[redacted]	[redacted]	[redacted]	[redacted]	[redacted]	[redacted]	[redacted]	Hispanic

1 - 8

Dialysis Connect

rmutell

Dialysis Connect

8

CURRENT HOSPITALIZATIONS

43

HOSPITALIZATIONS THIS MONTH

Find Patient

Current Hospitalizations [8]   Previous Hospita

Requested Documents

[redacted]

	Report	Date Uploaded
	Requested Information 1	11-FEB-2019 18:08
	Requested Information 2	11-FEB-2019 18:08
	Requested Information 3	11-FEB-2019 18:08
	Requested Information 4	11-FEB-2019 18:08
	Requested Information 5	11-FEB-2019 18:08
	Requested Information 6	11-FEB-2019 18:08
	Requested Information 7	11-FEB-2019 18:08
	Requested Information 8	11-FEB-2019 18:08

Close

Patient ID	Date Of Birth	Patient Race
[redacted]	[redacted]	Black / African American
		Hispanic
		White
		Hispanic
		Hispanic
		White
		White
		Hispanic

1 - 8

Communication Channel

Hospital and Dialysis Center

Dialysis Connect

rmutell

Home

Administration

Dialysis Connect

8
CURRENT HOSPITALIZATIONS

2
RE-ADMITTED PATIENTS

12
HOSPITALIZATIONS THIS WEEK

43
HOSPITALIZATIONS THIS MONTH

Find Patient

Current Hospitalizations [8] | Previous Hospitalizations

Go | Actions

	Patient ID	Clinic	Last Name	First Name	Gender	Social Security Number	Date Of Birth	Patient Race
	[redacted]							Black / African American
	[redacted]							Hispanic
	[redacted]							White
	[redacted]							Hispanic
	[redacted]							Hispanic
	[redacted]							White
	[redacted]							White
	[redacted]							Hispanic

1 - 8

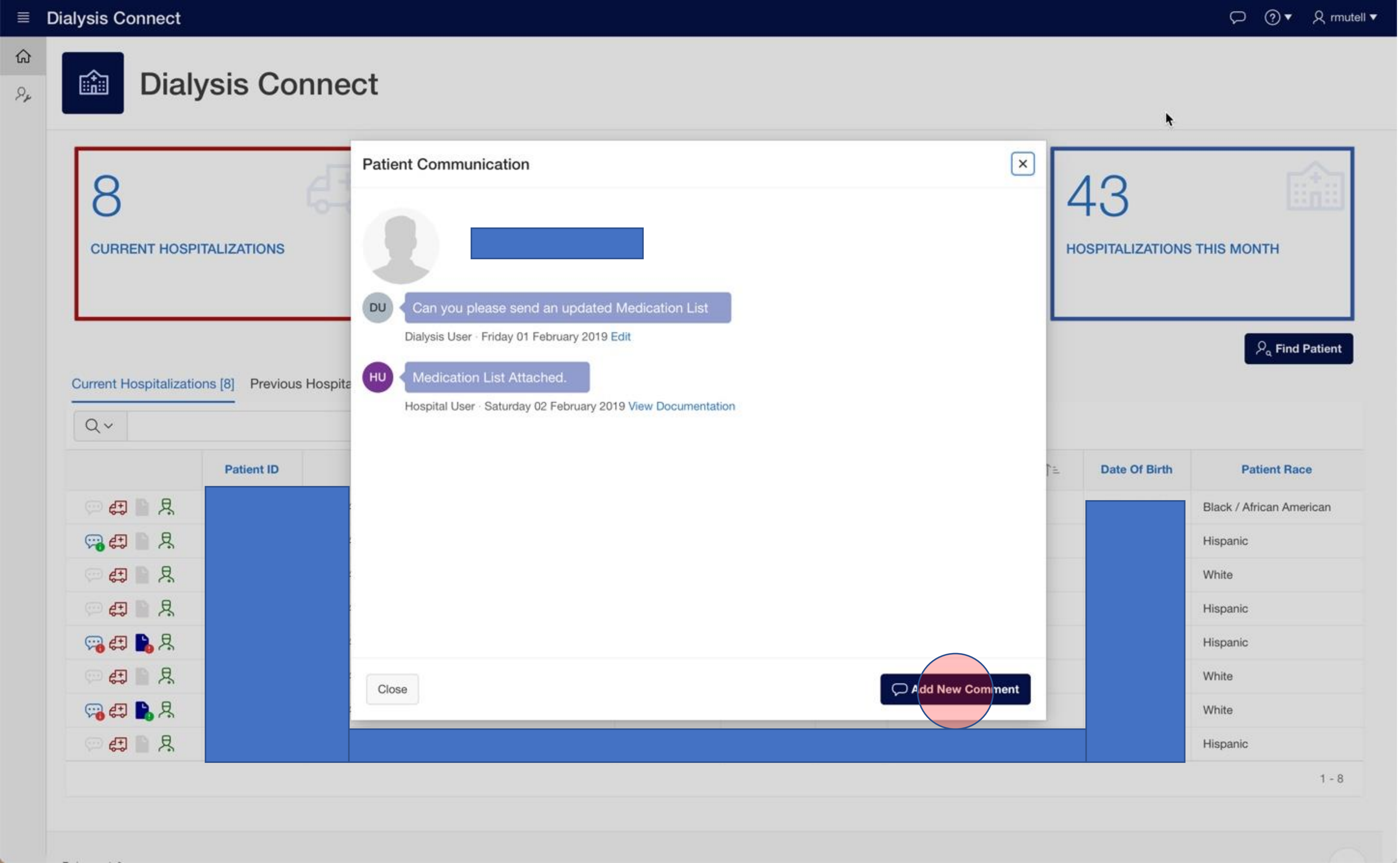
Dialysis Connect
rmutell
Dialysis Connect
8
CURRENT HOSPITALIZATIONS
43
HOSPITALIZATIONS THIS MONTH
Find Patient
Current Hospitalizations [8]
Previous Hospita
Patient ID
Date Of Birth
Patient Race
Black / African American
Hispanic
White
Hispanic
Hispanic
White
White
Hispanic
1 - 8
Patient Communication
DU
Can you please send an updated Medication List
Dialysis User · Friday 01 February 2019 Edit
HU
Medication List Attached.
Hospital User · Saturday 02 February 2019 View Documentation
Close
Add New Comment

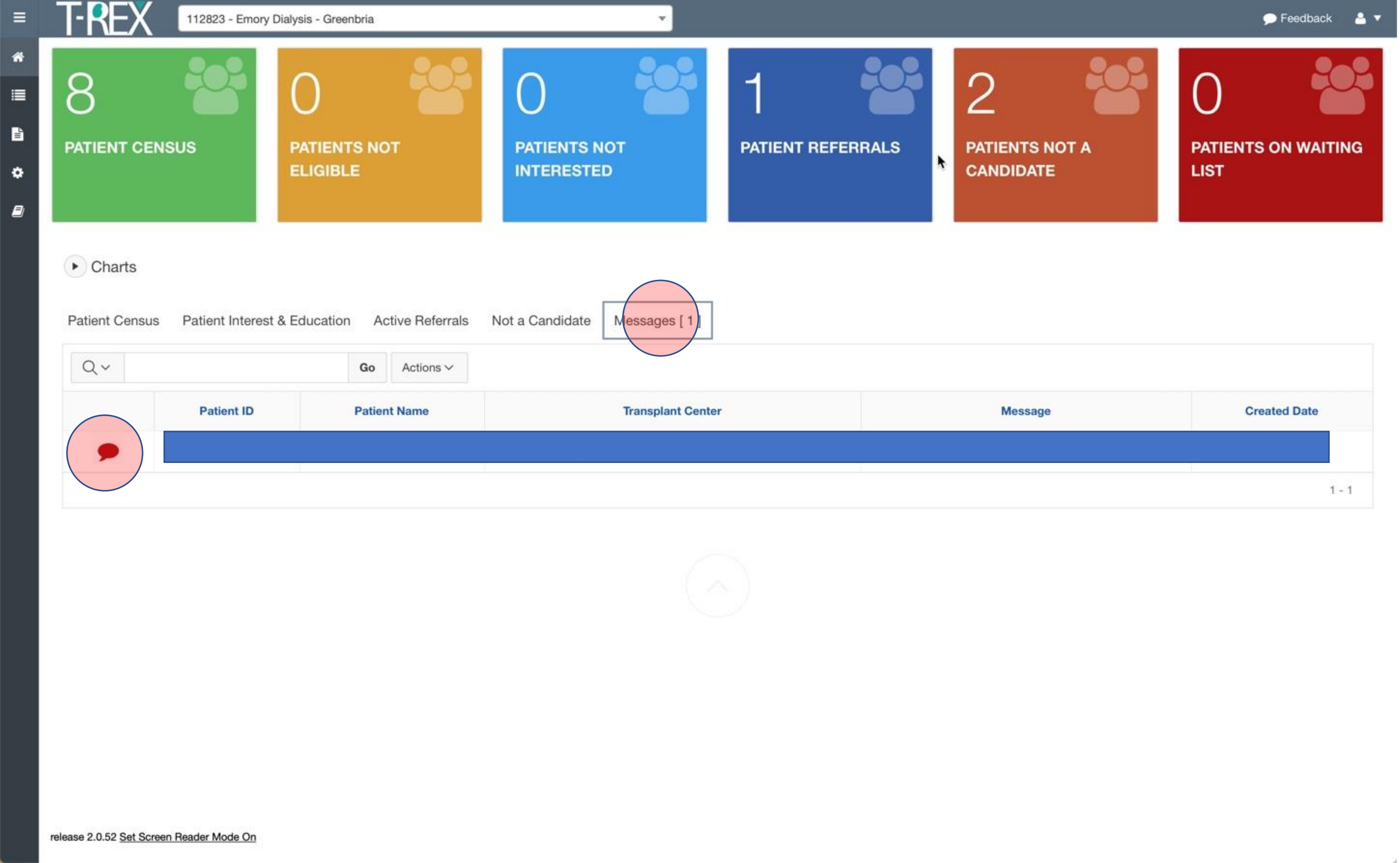
T-REX
112823 - Emory Dialysis - Greenbria
Feedback
8
PATIENT CENSUS
0
PATIENTS NOT ELIGIBLE
0
PATIENTS NOT INTERESTED
1
PATIENT REFERRALS
2
PATIENTS NOT A CANDIDATE
0
PATIENTS ON WAITING LIST
Charts
Patient Census
Patient Interest & Education
Active Referrals
Not a Candidate
Messages [1]
Go
Actions
Patient ID
Patient Name
Transplant Center
Message
Created Date
1 - 1
release 2.0.52 Set Screen Reader Mode On

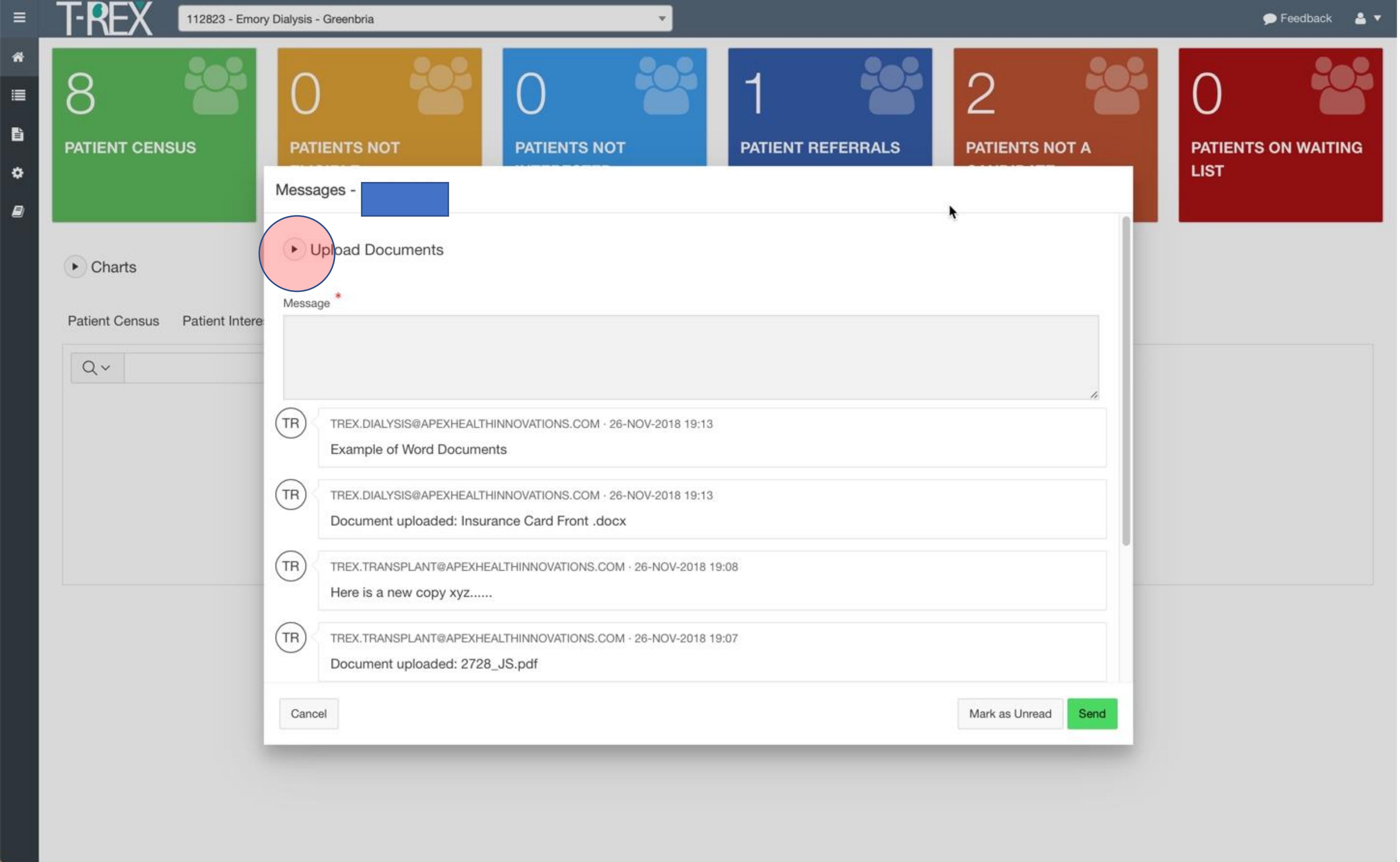
T-REX
112823 - Emory Dialysis - Greenbria
Feedback
8
PATIENT CENSUS
0
PATIENTS NOT
0
PATIENTS NOT
1
PATIENT REFERRALS
2
PATIENTS NOT A
0
PATIENTS ON WAITING LIST
Charts
Patient Census
Messages -
Upload Documents
Message *
TREX.DIALYSIS@APEXHEALTHINNOVATIONS.COM · 26-NOV-2018 19:13
Example of Word Documents
TREX.DIALYSIS@APEXHEALTHINNOVATIONS.COM · 26-NOV-2018 19:13
Document uploaded: Insurance Card Front .docx
TREX.TRANSPLANT@APEXHEALTHINNOVATIONS.COM · 26-NOV-2018 19:08
Here is a new copy xyz......
TREX.TRANSPLANT@APEXHEALTHINNOVATIONS.COM · 26-NOV-2018 19:07
Document uploaded: 2728_JS.pdf
Cancel
Mark as Unread
Send

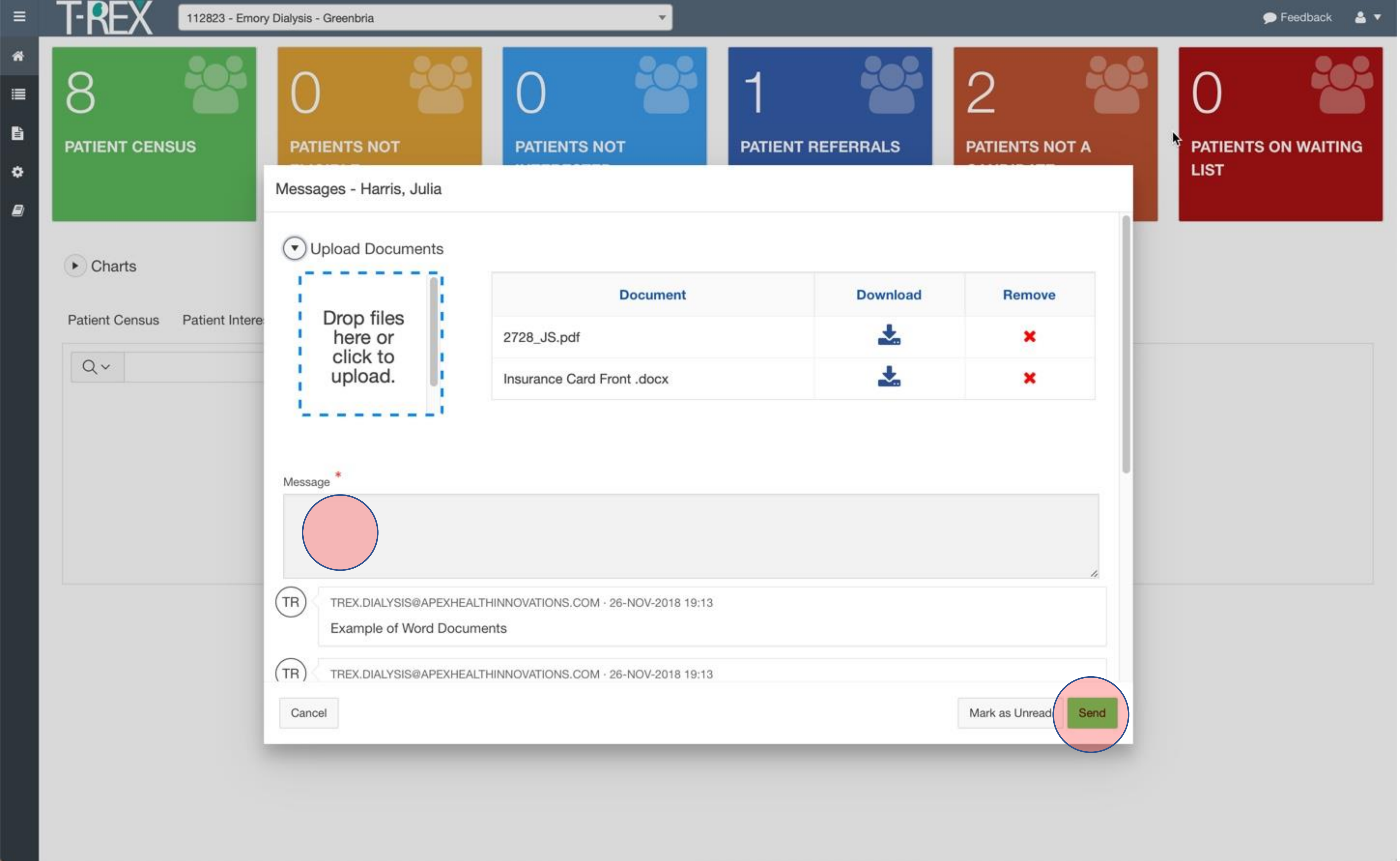

T-REX
112823 - Emory Dialysis - Greenbria
Feedback
8
PATIENT CENSUS
0
PATIENTS NOT
0
PATIENTS NOT
1
PATIENT REFERRALS
2
PATIENTS NOT A
0
PATIENTS ON WAITING LIST
Charts
Patient Census
Messages - Harris, Julia
Upload Documents
Drop files here or click to upload.
Document
Download
Remove
2728_JS.pdf
Insurance Card Front .docx
Message *
TR
TREX.DIALYSIS@APEXHEALTHINNOVATIONS.COM · 26-NOV-2018 19:13
Example of Word Documents
TR
TREX.DIALYSIS@APEXHEALTHINNOVATIONS.COM · 26-NOV-2018 19:13
Cancel
Mark as Unread
Send

Patient Discharged

Report to Dialysis Center

Dialysis Connect rmutell

Home
Administration

Dialysis Connect

8	2	12	43
CURRENT HOSPITALIZATIONS	RE-ADMITTED PATIENTS	HOSPITALIZATIONS THIS WEEK	HOSPITALIZATIONS THIS MONTH

Find Patient

Current Hospitalizations [8] | Previous Hospitalizations

Go | Actions

	Patient ID	Clinic	Last Name	First Name	Gender	Social Security Number	Date Of Birth	Patient Race
	[redacted]							Black / African American
								Hispanic
								White
								Hispanic
								Hispanic
								White
								White
								Hispanic

1 - 8

Dialysis Connect

rmutell

Discharge Report

[redacted]

Patient Information

Discharge Information

Discharge Diagnosis

Weight Reduction: Yes | **No**

Weight Loss Amount

Discharge Medicine

Discharge Summary Reports

Drop files here or click to upload.

Cancel

Send

8 CURRENT HOSPITALIZATIONS

Find Patient

Current Hospitalizations [8]

Patient ID	Date Of Birth	Patient Race
[redacted]	[redacted]	Black / African American
		Hispanic
		White
		Hispanic
		Hispanic
		White
		White
		Hispanic

1 - 8

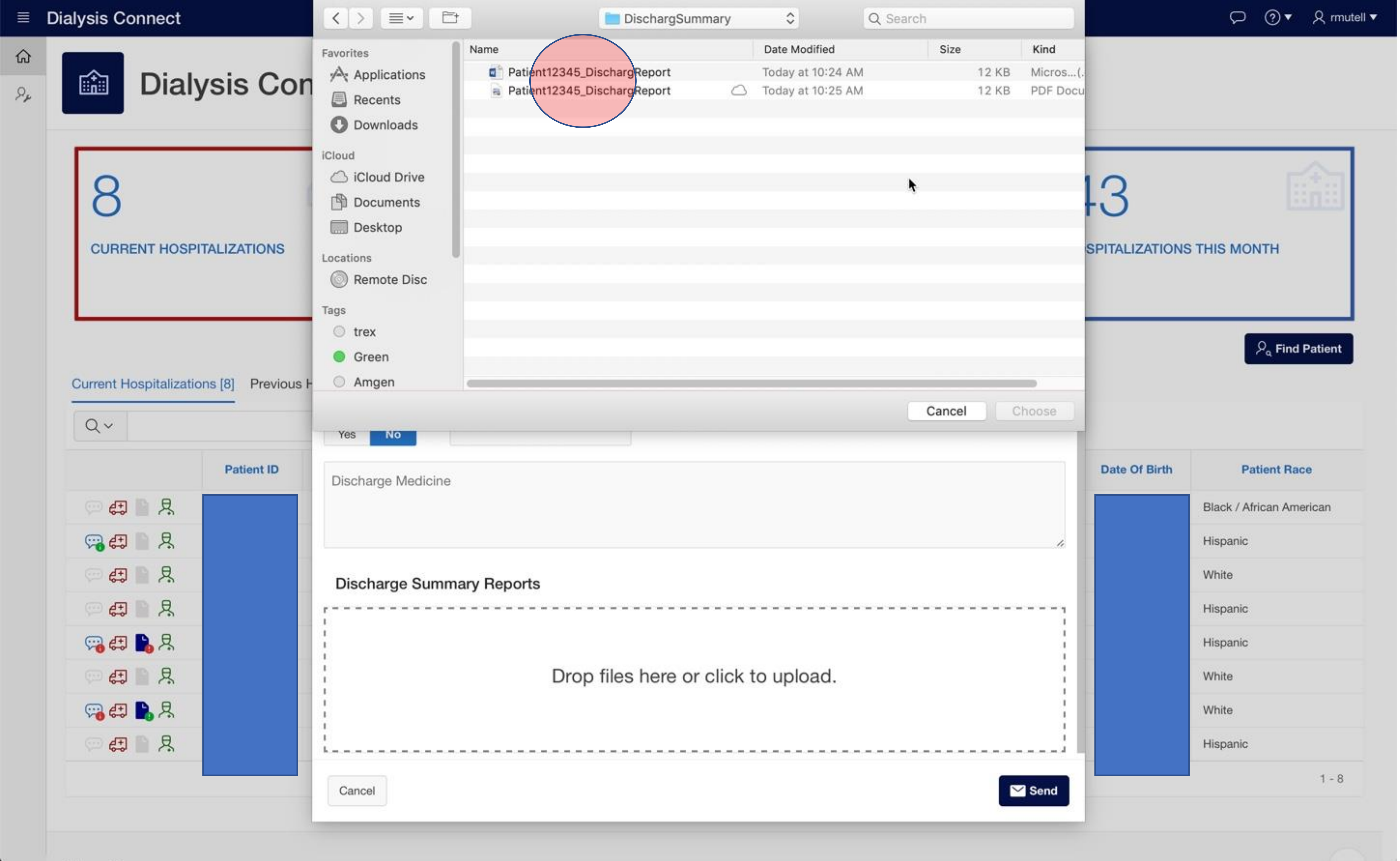
Dialysis Connect
rmutell
Dialysis Con
8
CURRENT HOSPITALIZATIONS
43
SPITALIZATIONS THIS MONTH
Find Patient
Current Hospitalizations [8]
Previous H
Patient ID
Date Of Birth
Patient Race
Black / African American
Hispanic
White
Hispanic
Hispanic
White
White
Hispanic
1 - 8
DischargSummary
Search
Favorites
Applications
Recents
Downloads
iCloud
iCloud Drive
Documents
Desktop
Locations
Remote Disc
Tags
trex
Green
Amgen
Name
Date Modified
Size
Kind
Patient12345_DischargReport
Today at 10:24 AM
12 KB
Micros...(.
Patient12345_DischargReport
Today at 10:25 AM
12 KB
PDF Docu
Cancel
Choose
Yes
No
Discharge Medicine
Discharge Summary Reports
Drop files here or click to upload.
Cancel
Send

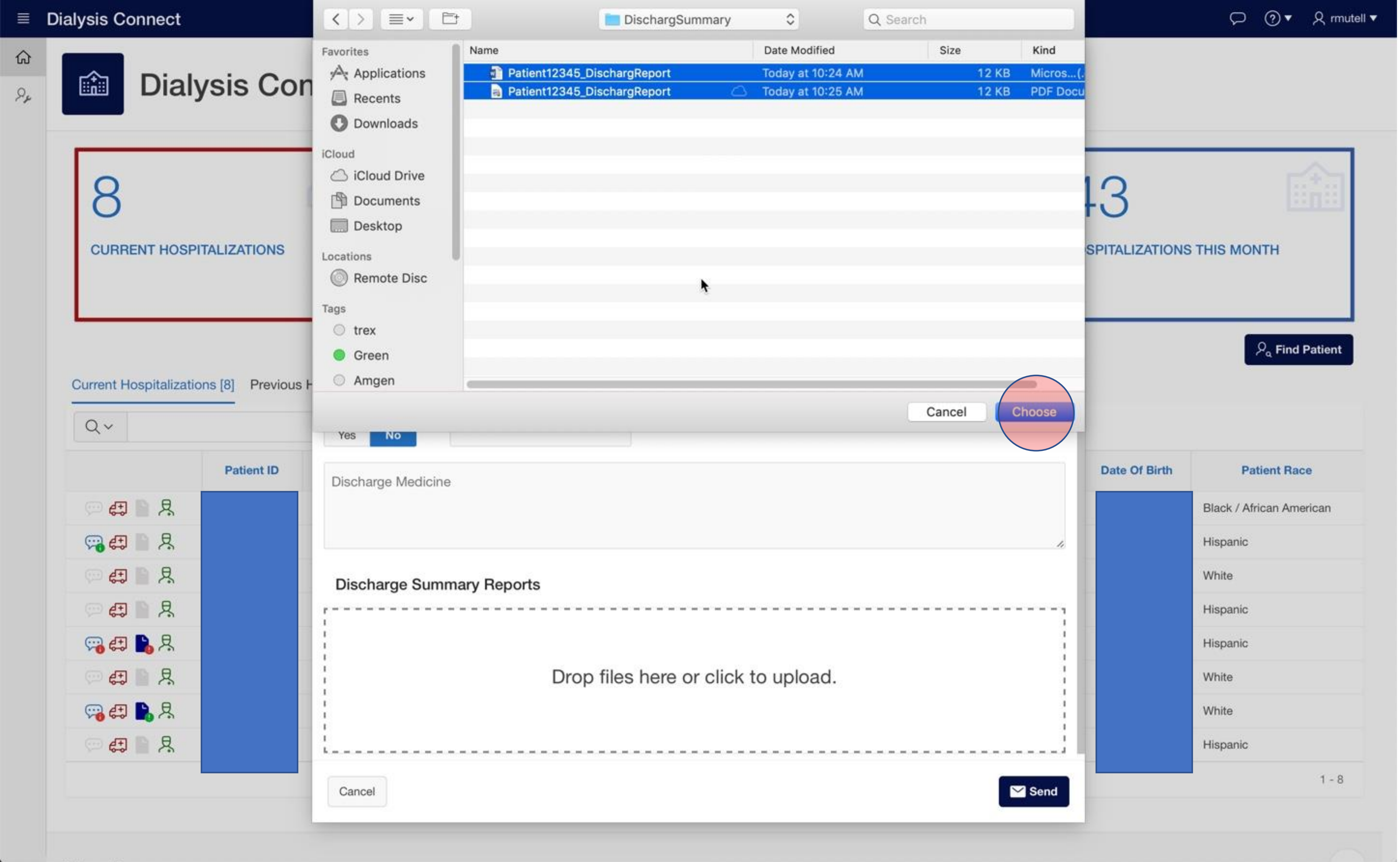
Dialysis Connect
DischargSummary
Search
rmutell
Favorites
Applications
Recents
Downloads
iCloud
iCloud Drive
Documents
Desktop
Locations
Remote Disc
Tags
trex
Green
Amgen
Name
Date Modified
Size
Kind
Patient12345_DischargReport
Today at 10:24 AM
12 KB
Micros...(.
Patient12345_DischargReport
Today at 10:25 AM
12 KB
PDF Docu
Cancel
Choose
Dialysis Con
8
CURRENT HOSPITALIZATIONS
43
SPITALIZATIONS THIS MONTH
Find Patient
Current Hospitalizations [8]
Previous H
Yes
No
Discharge Medicine
Discharge Summary Reports
Drop files here or click to upload.
Cancel
Send
Patient ID
Date Of Birth
Patient Race
Black / African American
Hispanic
White
Hispanic
Hispanic
White
White
Hispanic
1 - 8

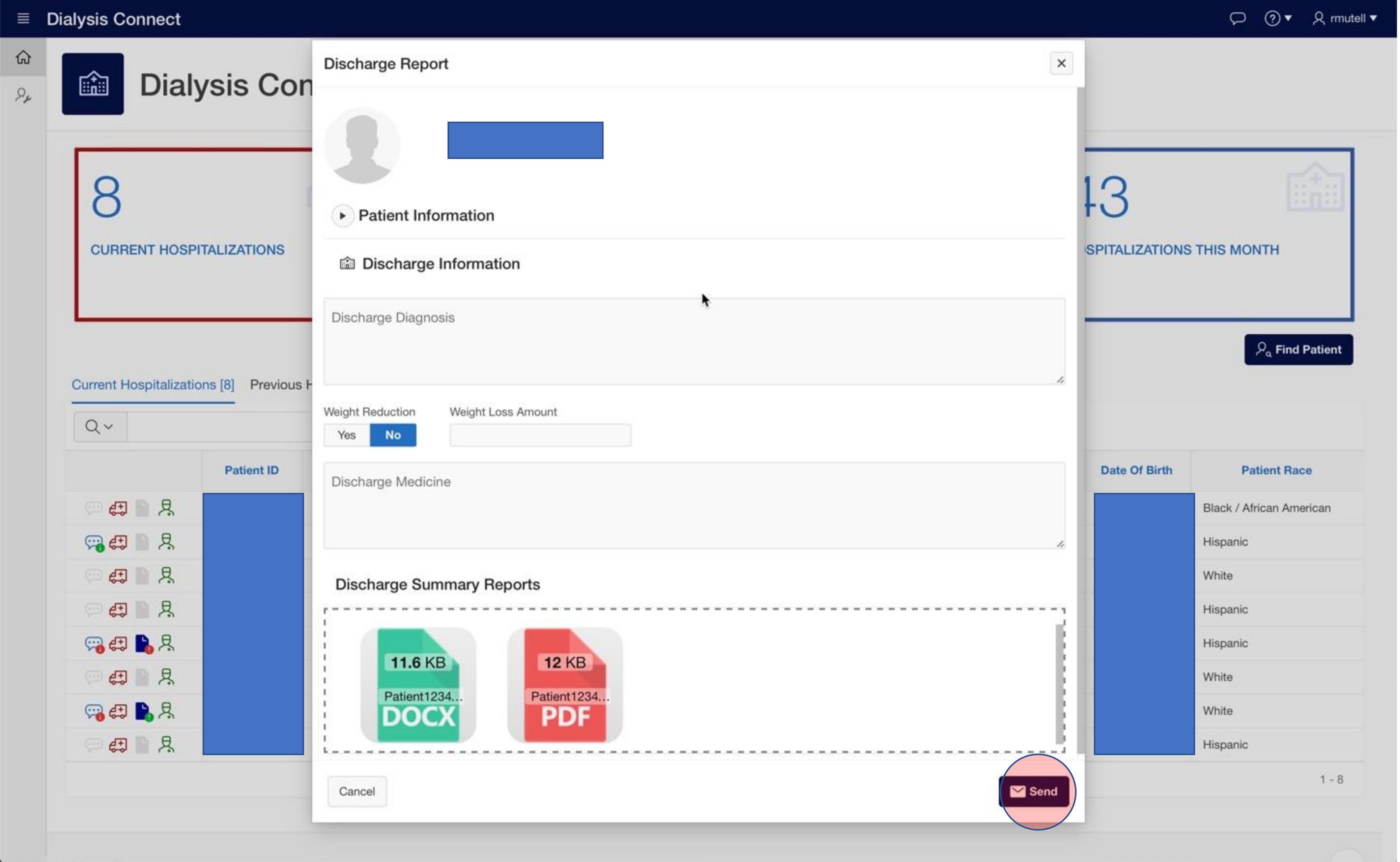
Dialysis Connect
rmutell
Dialysis Con
8
CURRENT HOSPITALIZATIONS
43
SPITALIZATIONS THIS MONTH
Find Patient
Current Hospitalizations [8]
Previous H
Patient ID
Date Of Birth
Patient Race
Black / African American
Hispanic
White
Hispanic
Hispanic
White
White
Hispanic
1 - 8
Discharge Report
Patient Information
Discharge Information
Discharge Diagnosis
Weight Reduction
Yes
No
Weight Loss Amount
Discharge Medicine
Discharge Summary Reports
11.6 KB
Patient1234...
DOCX
12 KB
Patient1234...
PDF
Cancel
Send

Notification Alerts

Email automatically sent to Nephrologist and Dialysis Staff
Track when Discharge Summary is selected in DialysisConnect
Reminders sent until Discharge Summary is viewed
