# Supplementary material for: Making sense of DialysisConnect: a qualitative analysis of stakeholder viewpoints on a web-based information exchange platform to improve care transitions between dialysis clinics and hospitals
Source: BMC Med Inform Decis Mak. 2021 Feb 9;21:47. doi: 10.1186/s12911-021-01415-y (PMC7871569; doi:10.1186/s12911-021-01415-y)
Supplement: Supplementary file 2 — Additional file 2: Table 1. Consolidated criteria for reporting qualitative research 32-item checklist for DialysisConnect coherence study [file 12911_2021_1415_MOESM2_ESM.docx]

| **Supplemental Table 1. Consolidated Criteria for Reporting Qualitative Research 32-item checklist** | |
| --- | --- |
| **Domain 1: Research team and reflexivity** | |
| 1. Interviewer/facilitator | Ann E. Vandenberg |
| 1. Credentials | PhD |
| 1. Occupation | Research Scientist |
| 1. Gender | Female |
| 1. Experience and training | A trained gerontologist with a focus on behavioral science and health education, Dr. Vandenberg is experienced with engaging diverse participants in dialogue about aspects of medical care including for studies exploring new technologies, workflow, care assessments, and health education interventions. |
| 1. Relationship established | Three nephrologist participants were in a previous research focus group led by the same facilitator and related to physical functioning assessment for dialysis patients. |
| 1. Participant knowledge of the interviewer | None other than her affiliation with Emory University School of Medicine Division of General Medicine and Geriatrics and role on study and any associated professional knowledge. |
| 1. Interviewer characteristics | Dr. Vandenberg used her first name and led the conversation around a conference table from a seated position, except when turning on DialysisConnect simulation from the podium. |
| **Domain 2: Study design** | |
| 1. Methodological orientation and theory | Grounded theory methods (Charmaz, 2014) and theoretical focused coding according to Normalization Process theory coherence construct components comparing within and across stakeholder groups to generate themes (Braun and Clarke, 2006). Thematic analysis can provide the foundation for a complete grounded theory analysis. |
| 1. Sampling | Purposively sampled from 4 target groups comprised of: 37 hospitalists at one hospital; 17 hospital staff (11 nurses, 5 social workers, and 1 pharmacist); 24 dialysis clinic nephrologists (12 nephrologists and 12 nephrology fellows) across 4 dialysis clinics; 120 dialysis clinic staff (85 dialysis nurses, 27 dialysis social workers, and 8 dieticians) across 4 dialysis clinics. |
| 1. Method of approach | Emails were sent to departmental email lists by the principal investigator inviting participation, with several follow up emails by the project coordinator. Participants who recruited into focus groups up to a maximum of 10 people. Participants were consented into the focus groups in person prior to the focus groups. |
| 1. Sample size | 31 participants, 4 focus groups |
| 1. Non-participation | 9 of those emailed declined to participate (1 nephrologist, 7 hospitalsts, and 1 hospital staff member; 1 hospitalist); the other non-participants were non-reponders. |
| 1. Setting of data collection | Focus groups were conducted in healthcare classrooms or conference rooms at the hospital (hospitalist and hospital staff groups); university (neprhologists); or one dialysis center (dialysis clinic staff). Dinner or breakfast was provided for these meetings. |
| 1. Presence of non-participants | At least one staff member or student observer attended, sitting away from the table who took notes during the discussion. |
| 1. Description of sample | See Table 2. |
| 1. Interview guide | See Table 1. |
| 1. Repeat interviews | N/A |
| 1. Audio/visual recording | Audio recorded. |
| 1. Field notes | The non-participant student or staff member(s) took field notes on nonverbal communication, group dynamics, and level of engagement. |
| 1. Duration | Focus groups lasted 90 minutes without a break. |
| 1. Data saturation | The scope of the current project and available resources precluded sampling until we reached theoretical saturation. Stated themes by coherence construct did repeat across groups and are displayed in Table 3. |
| 1. Transcripts returned | Time and resource constraints precluded sharing transcripts with participants for review. |
| **Domain 3: Analysis and findings** | |
| 1. Number of data coders | 1 |
| 1. Description of coding tree | Components of the Normalization Process Theory construct of coherence (differentiation, communal specification, individual specification, and internalization) and additional relevant codes (coherence of the problem, interview phase, and anticipatory internalization). |
| 1. Derivation of themes | During open coding, transcripts were coded using gerunds to describe each action statement in the transcript. Open codes were then examined and grouped through focused coding. Codes within components were then examined across stakeholder groups for concordances and discordances and grouped into themes. |
| 1. Software | Nvivo 12, QSR International. |
| 1. Participant checking | Member checks were included at points during the focus groups and at the end during summary discussion. In this fashion, opportunity was provided for participants to clarify, correct, or confirm the facilitator’s summary statements. Time and resource constraints precluded asking participants to provide feedback on the findings following analysis. |
| 1. Quotations presented | See Results and Table 3. |
| 1. Data and findings consistent | There is consistency between the data presented and the findings. |
| 1. Clarity of major themes | Themes are presented within the NPT construct components of differentiation, communal specification, individual specification, and internalization. Interaction between categories beyond these categories continued but is beyond the scope of this thematic analysis. |
| 1. Clarity of minor themes | N/A |
